# Review of the Chinese Leucospidae (Hymenoptera, Chalcidoidea)

**DOI:** 10.3897/zookeys.651.11235

**Published:** 2017-02-02

**Authors:** Xin-hai Ye, Cornelis van Achterberg, Qi Yue, Zai-fu Xu

**Affiliations:** 1 College of Agriculture and Biotechnology, Zhejiang University, Hangzhou 310058, China; 2 Department of Entomology, South China Agricultural University, Guangzhou 510640, China; 3 Shaanxi Key Laboratory for Animal Conservation, Northwest University, Xi’an 710069, China

**Keywords:** China, Leucospidae, *Leucospis*, new species, new record, Oriental region, Palaearctic region

## Abstract

The Chinese fauna of the family Leucospidae (Hymenoptera, Chalcidoidea) is reviewed and illustrated for the first time. Twelve species of *Leucospis* Fabricius, 1775 are recorded; of which two species are new to science: *Leucospis aequidentata* **sp. n.** and *Leucospis shaanxiensis* **sp. n.** and one species is reported new for China: *Leucospis intermedia* Illiger, 1807. An identification key to Chinese species is included. A lectotype is designated for *Leucospis aurantiaca* Shestakov, 1923.

## Introduction

Leucospidae is a small and rarely encountered family, including several of the largest species known in the Chalcidoidea (Bouček 1974a). Only four genera are recognized in the Leucospidae, *Leucospis* Fabricius, 1775, *Polistomorpha* Westwood, 1839, *Micrapion* Kriechbaumer, 1894, and *Neleucospis* Bouček, 1974 (Bouček 1974a; Noyes 2016). In the Palaearctic and Oriental regions the Leucospidae are represented by the genus *Leucospis* Fabricius with thirty-eight species. Thirteen species are known from the Palaearctic region, twenty-two from the Oriental region, and three species occur in both regions. Bouček (1974a) reported nine species from China, and no species have been added since his revision (Noyes 2016). Our knowledge of the distribution of Chinese Leucospidae is still far from complete despite the recent papers by Sheng (2003), He et al. (2004), Luo and Liu (2009), and Shen (2014). This study summarizes our knowledge of the recognition, composition and distribution of the *Leucospis* species in China. Twelve species of *Leucospis* are included in this study, of which two are new to science and described in this paper, and one species is new for China.

## Materials and methods

All specimens were examined and described using a Leica MZ125 stereomicroscope. Photographs of specimens from South China Agricultural University (SCAU) were taken with a CoolSNAP digital camera attached to a Zeiss Stemi 2000-CS stereomicroscope. Images were processed using Image-Pro Plus software. Primary types present in depositories outside China could be only studied by photos because of logistic reasons; the photos were kindly provided by Dr. Elijah Talamas (holotype of *Leucospis bakeri* Crawford, *Leucospis indiensis* Weld, *Leucospis orientalis* Weld, and *Leucospis ornatifrons* Weld), by Dr. Natalie Dale-Skey Papilloud (lectotypes of *Leucospis exornata* Walker and *Leucospis sinensis* Walker, and holotype of *Leucospis femoricincta* Bouček), by Dr. Lars Vilhelmsen (lectotype of *Leucospis gigas* Fabricius), by Dr. Toshiharu Mita (holotype of *Leucospis yasumatsui* Habu), and by Mr. Paolo Rosa (lectotype of *Leucospis aurantiaca* Shestakov). Figures were adjusted using Photoshop CC 2015.0.0, mostly to change size and background.

Morphological terminology mainly follows Bouček (1974a). Abbreviations used in the descriptions as follows: **F1**, **F2**, **F3**, etc.= flagellomeres 1, 2, 3, etc.; **MS (or MSP)** = malar space, the shortest distance between base of mandible and lower margin of compound eye; **OOL** = the shortest distance between posterior ocellus and compound eye; **POD** = posterior ocellus diameter; **POL** = the shortest distance between posterior ocelli; **T1, T2**, etc. = metasomal tergites 1, 2, etc.

Examined specimens are deposited in the following institutions:

BMNH
Natural History Museum, London, UK 


KYUN
 Entomological Laboratory, Kyushu University, Fukuoka, Japan 


RMNH
Naturalis Biodiversity Center, Leiden, the Netherlands 


SCAU
Hymenoptera Collection, South China Agricultural University, Guangzhou, China 


USNM
 Smithsonian National Museum of Natural History, Washington, DC, USA 


ZISP
Zoological Institute, Russian Academy of Sciences, St. Petersburg, Russia 


ZMUC
 Zoologiske Museum, University of Copenhagen, Denmark 


## Systematics

### Leucospidae

Taxon classificationAnimaliaHymenopteraLeucospidae

Walker, 1834

Leucopsidae Walker, 1834: 13. Type genus: Leucopsis Duméril, 1823 (= Leucospis Fabricius, 1775).
Leucospidae Walker: Haliday 1839: ii; Habu 1962: 165; Bouček 1974a: 9; Schmid-Egger 2010.

#### Diagnosis.

Body usually black or brown, with yellow, orange, reddish brown or whitish markings; antenna 13-segmented, F1 petiolate, without anellus, and no multiporous plate sensillae; tegula elongate, at least two times as long as broad, reaching pronotum or nearly so; forewing often longitudinally folded at rest; hind femur markedly swollen with one or more teeth ventrally; hind tibia strongly curved; ovipositor generally exserted and curved upward over metasoma (Bouček 1974a; Hanson 1995; Noyes 2016).

#### Biology.

Ectoparasitoids of solitary aculeate Hymenoptera, especially Megachilidae and Anthophoridae (Burks 1961; Habu 1962; Bouček 1974a; Cooperband et al. 1999; Schmid-Egger 2010).

#### Distribution.

Cosmopolitan.

#### Valid species.

139 species in four genera (Alexandre et al. 2013; Noyes 2016).

### Genus Leucospis

Taxon classificationAnimaliaHymenopteraLeucospidae

Fabricius, 1775

Figs 1–2 , 3–4 , 5 , 6–13 , 14 , 15–22 , 23–27 , 28, 29 , 30–33 , 34 , 35–42 , 43 , 44–51 , 52 , 53–60 , 61 , 62–70 , 71–73 , 74 , 75–83 , 84, 85 , 86, 87 , 88 , 89–95 , 96, 97 , 98 , 99–105 , 106, 107 , 108, 109 , 110 , 111–118 , 119, 120 , 121–123

Leucospis Fabricius, 1775: 361. Type species: Leucospis dorsigera Fabricius, 1775, by monotypy.
Leucospis Fabricius: Habu 1962: 169; Bouček 1974a: 28; Narendran 1986: 43; Madl and Schwarz 2012: 1222.

#### Diagnosis.

Clypeus with lower margin often emarginated and with a small median tooth; mandible bare at apex, always with strong lower tooth; maxillary and labial palpi 4 and 3 segments, respectively; OOL distinctly longer than POD; scutellum without cross-carina; dorsellum usually transverse, often carinate posteriorly, sometimes bidentate; propodeum often with median carina; hind coxa enlarged, sometimes with dorsal tooth or dorsal lobe; hind femur with basal ventral tooth often located before middle; hind tibia with outer spur always shorter than inner spur, frequently apex of hind tibia ventrally produced into a spine; female with T6 distinctly separated from epipygium and ovipositor curved upward; male with T2 always transverse (Bouček 1974a; Grissell and Schauff 1997).

#### Biology.

Ectoparasitoids of solitary aculeate Hymenoptera, mostly bees (Bouček 1974a; Schmid-Egger 2010).

#### Distribution.

Cosmopolitan (Madl and Schwarz 2012, 2014; Noyes 2016).

#### Valid species.

The genus contains 123 valid species, of which 119 are assigned to sixteen species groups (Bouček 1974a, 1974b; Habu 1977; Bouček and Narendran 1981; Naumann 1981; Engel 2002; Grissell and Cameron 2002; Pauly et al. 2003; Nagase 2007; Darling and Cardinal 2005; Pujade-Villar and Caicedo 2010; Schmid-Egger 2010; Genaro 2012; Noyes 2016). In this paper, twelve species are reported from China, including two species new to science and one new for China. The checklists and known distribution of *Leucospis* spp. in China are given in Table 1.

**Table 1.** Revised checklist and known distribution of *Leucospis* species in China. An asterisk indicates a new record.

Species	Distribution in China
*Leucospis aequidentata* sp. n.	*Fujian, *Guangdong, *Hubei, *Hunan
*Leucospis aurantiaca* Shestakov, 1923	Inner Mongolia
*Leucospis bakeri* Crawford, 1914	Taiwan
*Leucospis femoricincta* Bouček, 1974	*Guangdong, Macao
*Leucospis gigas* Fabricius, 1793	Beijing, Inner Mongolia
*Leucospis histrio* Maindron, 1878	Guangdong, *Hainan
*Leucospis intermedia* Illiger, 1807	*Xinjiang
*Leucospis japonica* Walker, 1871	Beijing, Guangdong, Guangxi, Guizhou, Hebei, Henan, Hong Kong, Hubei, *Hunan, Jiangsu, Jiangxi, Shaanxi, Shanghai, Shanxi, Sichuan, Taiwan, Yunnan, Zhejiang
*Leucospis petiolata* Fabricius, 1787	Fujian, *Guangdong, Hong Kong, Macao
*Leucospis shaanxiensis* sp. n.	*Shaanxi
*Leucospis sinensis* Walker, 1860	Jiangsu, Shanghai, Taiwan
*Leucospis yasumatsui* Habu, 1961	Shanxi

#### Key to the Chinese species of genus *Leucospis* Fabricius

**Table:** 

1	Hind femur with 3–4 long and slender teeth (apart from small apical teeth; Figs 87, 95, 105, 118, 119); basal tooth of hind femur smaller than following three teeth (Figs 87, 95, 105, 118, 119)	**2**
–	Hind femur with many small to medium-sized teeth (Figs 4, 13, 22, 33, 42, 51, 60, 70, 83); basal tooth of hind femur larger than following teeth or about as large (Figs 4, 13, 22, 33, 42, 51, 60, 70, 83)	**6**
2	Ovipositor sheath medium-sized, up to middle of T5 (Figs 114, 120); frons black (Fig. 115); T1 of ♂ truncate posteriorly; metasoma of ♂ distinctly constricted subbasally	**3**
–	Ovipositor sheath long, at least up to T1 (Figs 85, 92, 102); frons or frontovertex usually with yellow patch laterally (Figs 86, 93, 103); T1 of ♂ concave posteriorly; metasoma of ♂ slightly constricted subbasally	**4**
3	Malar space 0.17–0.19 times height of eye (about as long as width of F2); hind femur finely and sparsely punctate and with large smooth interspaces (Fig. 119); T1 of ♀ without ovipositorial furrow, but with median carina (Fig. 120); premarginal carina of pronotum usually distinct; anterior half of T5 black (Fig. 120)	***Leucospis sinensis* Walker**
–	Malar space 0.22–0.30 times height of eye (Fig. 115; at least 1.2 times as long as width of F2); hind femur finely and densely punctate and with small smooth interspaces (Fig. 118); T1 of ♀ without ovipositorial furrow or carina medially (Fig. 114); premarginal carina of pronotum indistinct and replaced by a raised but blunt rib (Fig. 112); anterior half of T5 of Chinese specimens reddish brown (Fig. 114)	***Leucospis petiolata* Fabricius**
4	Dorsellum rounded posteriorly (Fig. 90); anterior ridge separating double ovipositorial furrow broad and coarsely punctate (Figs 90, 92); hind coxa partly impunctate medio-posteriorly (Fig. 91)	***Leucospis histrio* Maindron**
–	Dorsellum bidentate or concave posteriorly (Fig. 102); at most with a narrow and smooth anterior ridge separating double ovipositorial furrow (Fig. 102); hind coxa usually without impunctate area (Fig. 101)	**5**
5	F2–F4 of ♀ broader than long (Fig. 99; also in ♂); clypeus at most moderately protruding ventrally (Fig. 103); ovipositor sheath at least reaching anterior margin of T1 (Fig. 102)	***Leucospis intermedia* Illiger**
–	F2–F4 of ♀ distinctly longer than broad (also in ♂); clypeus conspicuously protruding ventrally (Fig. 86); ovipositor sheath usually not reaching anterior margin of T1 (Figs 84, 85)	***Leucospis gigas* Fabricius**
6	T1 of ♀ without ovipositorial furrow medially (Figs 2, 107); ovipositor sheath up to basal third of T5 (Figs 2, 107); hind tibia truncate apically, without apical spine (Fig. 4); body largely pale orange or reddish brown (Figs 1, 2, 106, 107)	**7**
–	T1 of ♀ with ovipositorial furrow medially (Figs 9, 29, 38, 56, 85); ovipositor sheath at least extending up to T1 (Figs 9, 29, 38, 56, 85); body largely black with yellow or orange pattern (Figs 5, 14, 28, 34, 43, 52, 61, 74); hind tibia protruding ventro-apically, with distinct apical spine (Figs 33, 42, 51, 61, 79, 83)	**8**
7	Hind leg entirely orange without any pattern (Figs 1, 4); hind femur shiny and sparsely pubescent (Fig. 4); mesoscutum bicoloured (orange with black pattern; Fig. 2); ovipositorial furrow up to anterior margin of T1 (Fig. 2); dorsellum distinctly bifurcate dorso-posteriorly (Fig. 2)	***Leucospis aurantiaca* Shestakov**
–	Hind leg reddish brown to blackish brown, with yellow pattern (Fig. 106); hind femur mostly dull and densely pubescent (Fig. 106); mesoscutum tricoloured, black with yellow and reddish brown patches (Fig. 107); ovipositorial furrow up to middle of T5 (Fig. 107); dorsellum posteriorly with evenly curved carinae, weakly protruding dorso-posteriorly (Fig. 107)	***Leucospis bakeri* Crawford**
8	Discal carina of pronotum absent or indistinct and not angularly raised medially (Figs 5, 7, 14, 16, 28, 31); premarginal and marginal carinae usually less conspicuous (Figs 5, 7, 31)	**9**
–	Discal carina of pronotum distinct and subangularly raised medially (Figs 34, 36, 43, 46, 61, 77); premarginal and marginal carinae conspicuously developed (Figs 34, 43, 74)	**10**
9	T1 of ♀ with very shiny broad and convex ridge between double ovipositorial furrow anteriorly (Fig. 9); hind femur mainly black and with large and more or less lunate yellow patch (Figs 5, 13); scape yellow ventrally (Figs 6, 10, 11); ovipositor sheath reaching dorsellum (Figs 8, 9)	***Leucospis japonica* Walker**
–	T1 of ♀ ovipositorial furrow single, without shiny convex ridge anteriorly (Fig. 29); hind femur mainly blackish brown, with small obscure yellowish patch apico-dorsally (Figs 28, 33); scape blackish brown ventrally (Fig. 32); ovipositor sheath up to anterior margin of T1 (Fig. 29)	***Leucospis yasumatsui* Habu**
10	T1 in lateral view steep anteriorly, almost rectangularly protruding (indicated by arrow in Figs 34, 74), distinctly above dorsal level of mesosoma and in dorsal view with deep ovipositorial furrow medio-dorsally (Figs 38, 78); area next to ovipositorial furrow with smooth interspaces between moderately coarse punctures broad (mostly about equal to diameter of puncture or somewhat broader); ovipositorial furrow distinctly impressed on T4 (Figs 38, 78); flagellomeres of ♀ (except F1) hardly narrowed basally, F2–F4 of ♀ distinct longer than broad (Figs 35, 75); hind femur with an elongate triangular yellow patch ventrally (Figs 42, 51, 74, 83)	**11**
–	T1 in lateral view gradually lowered anteriorly (indicated by arrow in Fig. 52), near dorsal level of mesosoma and in dorsal view with shallower ovipositorial furrow medio-dorsally (Fig. 56); area next to ovipositorial furrow with rather narrow smooth interspaces between very coarse punctures (mostly narrower than diameter of puncture); ovipositorial furrow rather shallowly impressed on T4 (Fig. 56); flagellomeres of ♀♂ narrowed basally, F2–F5 of both sexes broader than long or as long as broad (Figs 53, 54, 62); hind femur with a lunate yellow mark subbasally (Figs 52, 60, 61, 70)	***Leucospis femoricincta* Bouček**
11.	Concavity below apical spine of hind tibia with slender spines and long setae (indicated by arrow in Fig. 83); mesoscutum with a pair of obscure yellow spots submedially and a pair of yellow stripes laterally (Figs 76, 77); teeth of hind femur forming a rather irregular row (Figs 74, 83); yellow band of T5 of ♀ in lateral view distinctly broader than apical black band (Figs 74, 78)	***Leucospis shaanxiensis* sp. n.**
–	Concavity below apical spine of hind tibia with rather robust spines and shorter setae (indicated by arrow in Fig. 51); mesoscutum without yellow patch laterally or yellow spots submedially (Figs 37, 46); teeth of hind femur forming a regular row (Figs 42, 43, 51); yellow band of T5 of ♀ in lateral view at most about as broad as apical black band (Figs 34, 38)	***Leucospis aequidentata* sp. n.**

### The *dorsigera*-group

**Diagnosis.** Marginal and premarginal carinae on pronotum distinct but not strongly recurved; basal tooth on hind femur at least as large as femoral teeth; propodeum short, not distinctly longer than dorsellum (Darling and Cardinal 2005).

#### Leucospis aurantiaca

Taxon classificationAnimaliaHymenopteraLeucospidae

Shestakov, 1923

Figs 1–2 , 3–4

Leucospis aurantiaca Shestakov, 1923: 96; Bouček 1974a: 196; Bouček and Narendran 1981: 12.

##### Type material.

Lectotype here designated, ♀ (ZISP), “CHINA, [Inner Mongolia], Alashan, oas[is] / Dyn-yuan-in, 18.VI.1908, P. Kozlov”, “*Leucospis aurantiaca* Shestakov”, “Lectotype”.

##### Diagnosis.

**Figures 1, 2.**
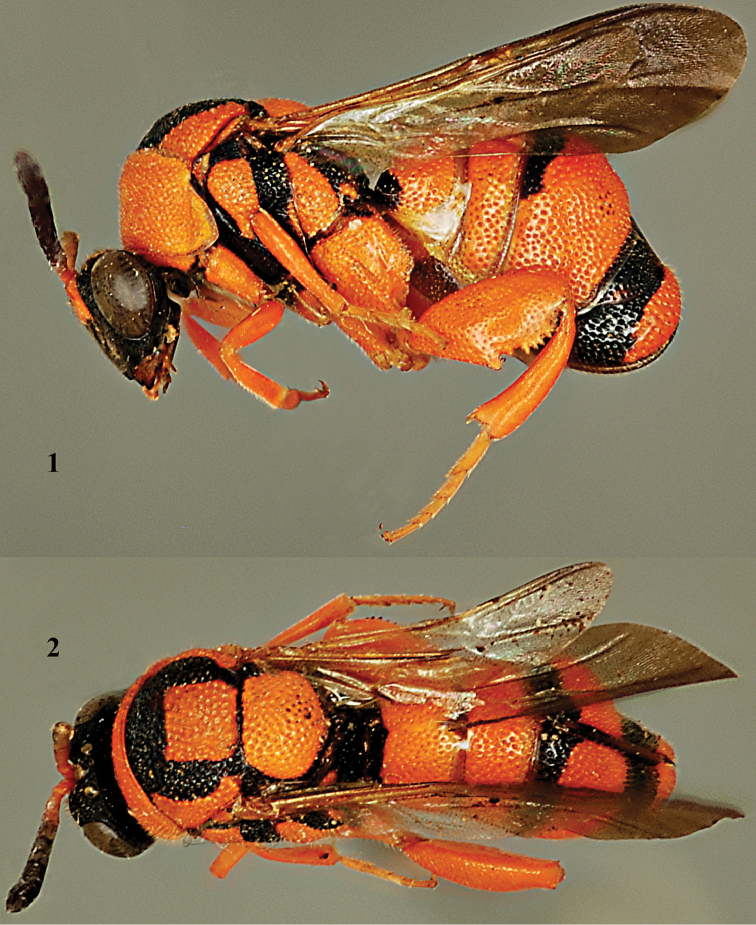
*Leucospis aurantiaca* Shestakov, ♀, lectotype. **1** Habitus lateral **2** habitus dorsal (courtesy of Mr. Paolo Rosa).

*Female*. Body mainly orange, with exception of orange brown to brown antennal flagellum, reddish brown mandible, black head, with black inverted U-shaped marking on mesoscutum, mesoscutellum with axillae black, black mesepimeron and propodeum, anterior margin of T1, anterior margin and posterior margin of T5, T6 and lower part of epipygium black, wings brownish, ovipositor sheath reddish brown (Fig. 2); propodeum raised medially, with weak median carina; hind femur with nine teeth ventrally, basal one largest (Fig. 4); metasoma strongly convex dorsally and medially (Fig. 1); T1 without ovipositorial furrow (Fig. 2); ovipositorial furrow up to black anterior margin of T5 (Fig. 2). *Male*. Unknown.

**Figures 3–4.**
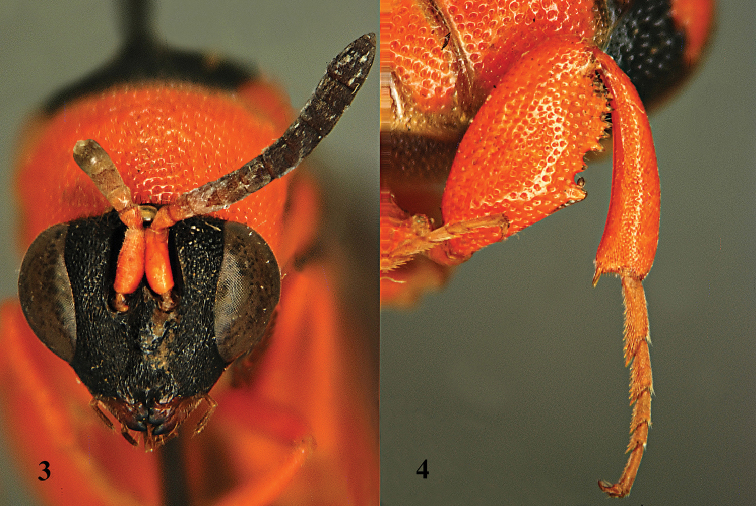
*Leucospis aurantiaca* Shestakov, ♀, lectotype. **3** Head frontal **4** hind leg (courtesy of Mr. Paolo Rosa).

##### Biology.

Unknown. Collected in June.

##### Distribution.

China (Inner Mongolia).

##### Remarks.

Bouček’s (1974a) interpretation was correct, despite he had no access to the type series.

#### Leucospis japonica

Taxon classificationAnimaliaHymenopteraLeucospidae

Walker, 1871

Figs 5 , 6–13 , 14 , 15–22 , 23–27

Leucospis japonica Walker, 1871: 56; Strand 1911: 98; Weld 1922: 28; Habu 1962: 170; Bouček 1974a: 194; Bouček and Narendran 1981: 9; Narendran 1986: 44; He et al. 2004: 104; Shen 2014: 1009.
Leucospis exornata Walker, 1871: 57. Syn. by Bouček (1974a).
Leucospis japonica var. formosana Strand, 1911: 98. Syn. by Bouček (1974a).
Leucospis orientalis Weld, 1922: 28. Syn. by Bouček (1974a).

##### Type material.

Lectotype of *Leucospis exornata*, ♀ (BMNH), “[CHINA], Hong Kong”, “B.M. Type Hym. 5.82”, NHMUK010370219, designated by Bouček (1974a). Holotype of *Leucospis orientalis*, ♀ (USNM), “CHINA, [Jiangsu], Soochow”, “Type No. 24388, U. S. N. M.”, USNMENT01223650. **Additional material**. 1♀, CHINA, Henan, Baotianman National Nature Reserve, 19.VIII.2007, Zai-fu Xu, No. 2016003689 (SCAU); 1♀, CHINA, Shaanxi, Huoditang, 5.VIII.2015, Jun Feng (SCAU), No. 2016000033; 2♀, same locality and date, Yi-cheng Li, No. 2016000032, 2016000032 (SCAU); 3♀1♂, Shaanxi, Liuba, 7.VIII.2015, Zi Hou, No. 2016000013–2016000015, 201600049 (SCAU); 3♀, same locality and date, Yi-cheng Li, No. 2016000016–2016000018 (SCAU); 1♀, same locality and date, Jun Feng, No. 2016000021 (SCAU); 1♂, Shaanxi, Taibai, 10.VIII.2015, Jun Feng, No. 2016000029 (SCAU); 1♀1♂, same locality and date, Zi Hou, No. 2016000019, 2016000028 (SCAU); 2♀1♂, same locality and date, Yi-cheng Li, No. 2016000025, 2016000026, 2016000027 (SCAU); 2♀4♂, CHINA, Jiangxi, Nanchang, Xiangshan Forest Park, 11–16.VII.2016, Hu-ting Zhou, No. 2016000139–2016000144 (SCAU); 1♂, CHINA, Hubei, Huanggang, 8.VII.2009, Chun-hong Zheng, No. 2016002957 (SCAU); 14♀34♂, CHINA, Hubei, Jingshan, 15.VII.2009, Yuan Ye, No. 2016000001–2016000041, 2016000043–2016000049 (SCAU); 4♀1♂, CHINA, Hunan, Hupingshan National Nature Reserve, 10–13.VII.2009, Qing-hui Yi, No. 2016000069, 2016000071, 2016000073, 2016000076, 2016000077 (SCAU, RMNH); 2♀1♂, CHINA, Hunan, Hupingshan National Nature Reserve, 14.VII.2009, Qing-hui Yi, No. 2016003691–2016003693 (SCAU); 24♀2♂, same locality, 8–12.VII.2009, Shi-hong Wang, No. 2016000086, 2016000087, 2016000089, 2016000092–2016000096, 2016000099, 2016000105, 2016000111, 2016000114, 2016000115, 2016000119, 2016000145, 2016000147, 2016000152, 2016000154, 2016000167, 2016000186, 2016000187, 2016000189, 2016000191–2016000195 (SCAU, KYUN, USNM, ZISP, ZMUC); 1♀1♂, same locality, 9.VII.2009, Qi Yang, No. 2016003687, 2016003688 (SCAU); 11♀3♂, same locality, 11–13.VII.2009, Qi Yang, No. 2016000216–2016000229 (SCAU); 2♂, same locality, 11–13.VII.2009, Xin Yuan, No. 2016000083, 2016000084 (SCAU); 1♀, same locality, 9.VII.2009, Ya-li Tang, No. 2016000125 (SCAU); 4♀1♂, CHINA, Hunan, Hengnan, Shanxi, 20.VIII–20.VII.2016, Yi-cheng Li, No. 2016002456–2016002458, 2016002473, 2016002474 (SCAU); 3♀1♂, CHINA, Hunan, Hengnan, Shanxi, 20.VIII–20.VII.2016, Zhi-neng Huang, No. 2016002459–2016002462 (SCAU); 2♀3♂, CHINA, Hunan, Hengnan, Shanxi, 20.VIII–20.VII.2016, Hu-ting Zhou, No. 2016002463–2016002466 (SCAU); 4♀2♂, CHINA, Hunan, Hengnan, Shanxi, 20.VIII–20.VII.2016, Shuang-shuang Li, No. 2016002467–2016002472 (SCAU); 1♀, CHINA, Guangdong, Meizhou, Meixian, 14–29.VII.2006, Cui-hong Xie & Wei-xin Xie, No. 2016000042 (SCAU); 1♀1♂, CHINA, Guangdong, Fogang, Guangyinshan Provincial Nature Reserve, 15–16.IX.2007, Zai-fu Xu, No. 2016000034, 2016000035 (SCAU); 1♀, CHINA, Guangdong, Huizhou, Xiangtoushan National Nature Reserve, 4.VI.2016, Zai-fu Xu, No. 2016000133 (SCAU); 1♀1♂, same locality and date, Qi Yue, No. 2016000136, 2016000138 (SCAU); 2♂, same locality and date, Zhi-neng Huang, No. 2016000134, 2016000135 (SCAU); 2♀1♂, CHINA, Guangdong, Huaiji, 22–23.X.2007, Zai-fu Xu, No. 2016000038–2016000040 (SCAU); 1♀, CHINA, Guangdong, Conghua, Xitoucun, 15.V.2016, Xiao-ya Wu, No. 2016003690 (SCAU); 1♀, CHINA, Guangxi, Mulun National Nature Reserve, 20.VII.2015, Zi Hou, No. 2016000030 (SCAU); 12♀, CHINA, Guizhou, Mayanghe, 27.IX–2.X.2007, Cui-hong Xie, No. 2016000001–2016000012 (SCAU); 6♀10♂, CHINA, Guizhou, Panxian, Machangxiang, 19.VII–6.VIII.2006, Zai-fu Xu, No. 2016000050–2016000055, 2016000057–2016000066 (SCAU).

##### Diagnosis.

**Figure 5.**
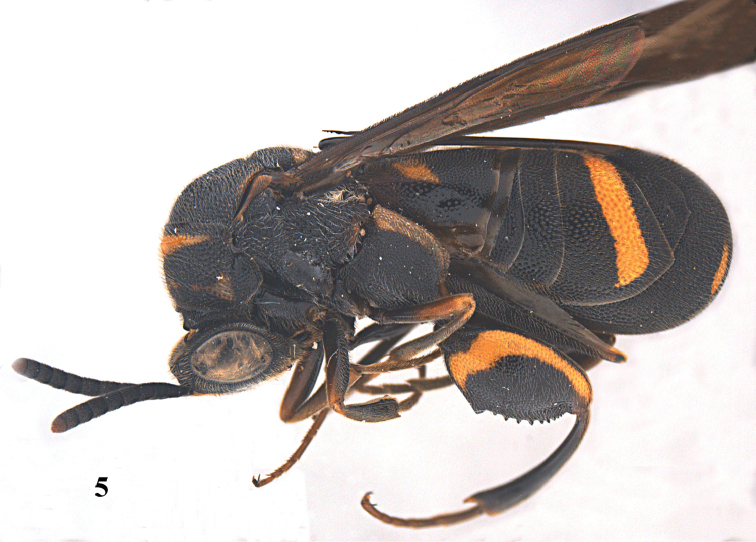
*Leucospis japonica* Walker, ♀ from Guangxi. Habitus lateral.

Body mainly black (Fig. 5), with exception of ventrally yellow antennal scape, pronotum with yellow transverse stripe anteriorly which sometimes is changed to three or six yellow spots, and yellow transverse stripe posteriorly which sometimes is triangular, mesoscutellum with pale yellow transverse band posteriorly, wings brownish, hind coxa pale yellow dorsally, hind femur with subbasal yellow lunate mark, from base crossing to dorsal border, T1 with a pair of large yellow spots laterad of ovipositorial furrow, T5 with broad yellow band near posterior margin, epipygium with a pair of yellow small spots postero-laterally (Figs 5, 9); propodeum raised medially and emarginated posteriorly, median carina weak or absent; hind femur with twelve teeth ventrally, basal one largest (Fig. 13); hind tibia produced into a spine ventro-apically (Fig. 5); T1 with ovipositorial furrow (Fig. 9); ovipositor sheath at least reaching posterior margin of dorsellum (Figs 5, 8, 9).

**Figures 6–13.**
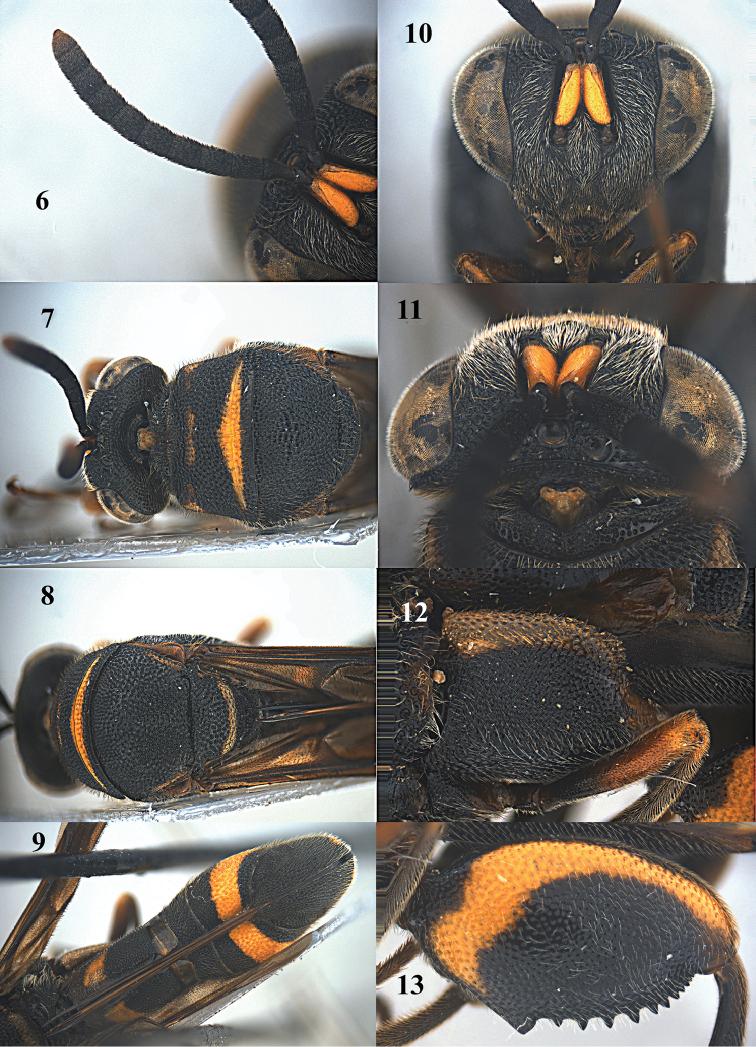
*Leucospis japonica* Walker, ♀ from Guangxi. **6** Antenna **7** pronotum and mesoscutum dorsal **8** mesosoma dorsal **9** metasoma dorsal **10** head frontal **11** head dorsal **12** hind coxa. **13** hind femur.

**Figure 14.**
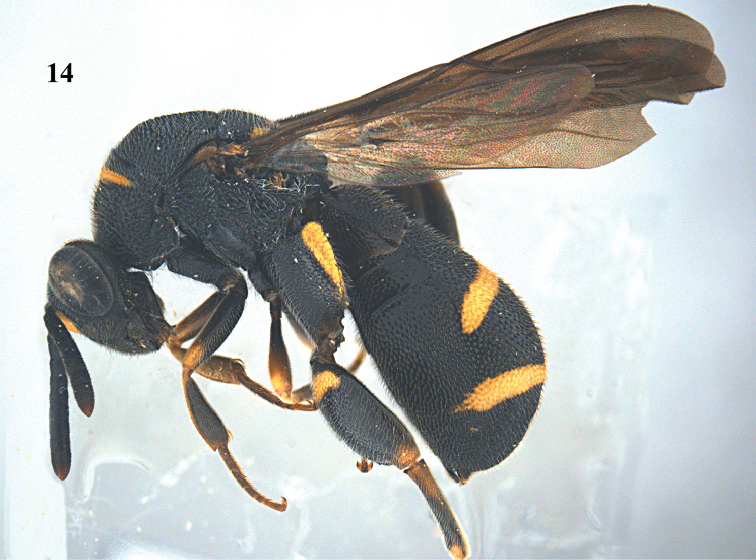
*Leucospis japonica* Walker, ♂ from Guangdong, Guangyinshan. Habitus lateral.

**Figures 15–22.**
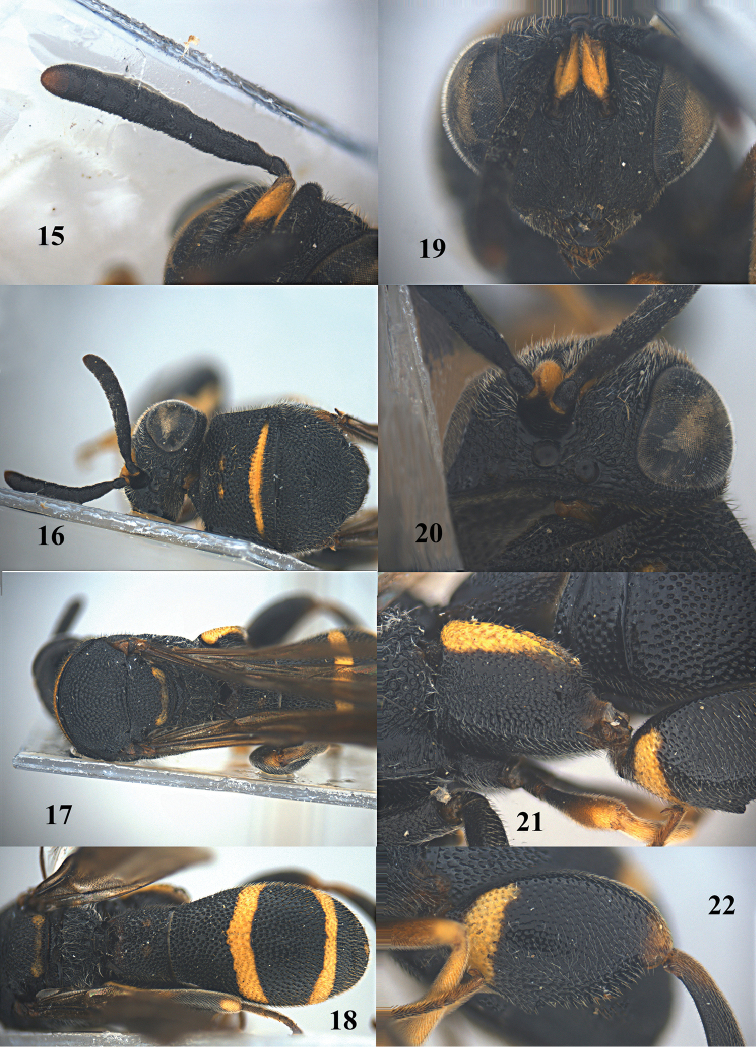
*Leucospis japonica* Walker, ♂ from Guangdong, Guangyinshan. **15** Antenna **16** head and pronotum dorsal **17** mesosoma dorsal **18** propodeum and metasoma dorsal **19** head frontal **20** head latero-dorsal **21** hind coxa **22** hind femur.

**Figures 23–27.**
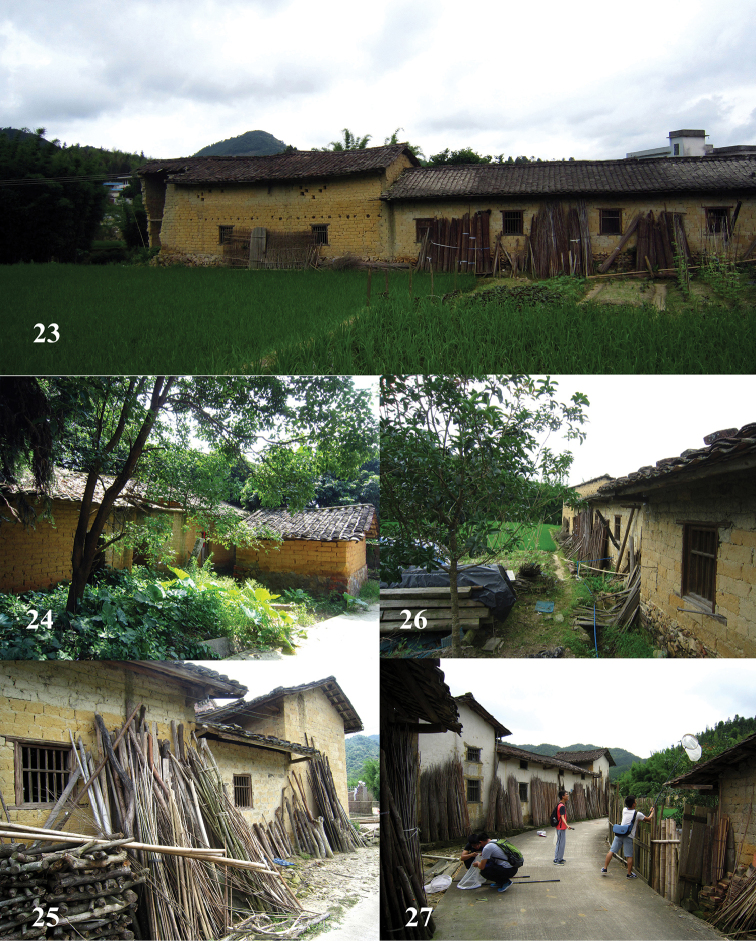
Habitats of *Leucospis japonica* Walker in China.

##### Redescription.

*Female*. Body length 8.0–14.0 mm. OOL = 2.1 POD; POL = 3.2 POD; MS = 1.9 POD.

*Head*. Coarsely and densely punctate, with dense short pubescence (Figs 10, 11). Occipital carina developed, sharp.

*Mesosoma*. Pronotum, mesoscutum, mesoscutellum, dorsellum, mesopleuron, metapleuron and propodeum coarsely and densely punctate with short pubescence (Figs 5, 7, 8). Pronotum with discal carina indistinct or weak, premarginal carina developed (Figs 5, 7). Dorsellum rounded and carinate posteriorly. Hind coxa with dense and moderate-sized punctures (but less densely antero-dorsally), and with short pubescence, postero-dorsally lamelliform (Fig. 12). Hind femur with twelve teeth ventrally, basal tooth largest (Fig. 13). Hind tibia produced into a spine ventro-apically (Fig. 5).

*Metasoma*. Metasoma with dense and medium-sized punctures, and with short pubescence. T1 narrower than T4 or T5 in dorsal view. T1 longest and T2 shortest. T1 with smooth double ovipositorial furrow, the latter subdivided by convex, broad and very shiny ridge. Ovipositor sheath long, at least reaching posterior margin of dorsellum (Figs 8, 9).

*Colouration*. Head black (Figs 10, 11). Antenna black, with scape yellow usually only ventrally (Figs 6, 10). Pronotum with yellow transverse stripe anteriorly, and yellow transverse stripe posteriorly (Fig. 7). Mesoscutum black, with obscure pale yellow stripe laterally (Fig. 8). Mesoscutellum black, with pale yellow transverse band posteriorly (Fig. 8). Wings brownish (Fig. 5). Hind coxa black, with indistinct yellow band dorsally (Figs 5, 12). Hind femur subbasally with yellow lunate mark from base crossing to dorsal border (Fig. 13). Metasoma black, T1 with a pair of big yellow spots laterad of ovipositorial furrow, T5 with broad yellow band near posterior margin (Fig. 9). Epipygium with a pair of small spots postero-laterally (Figs 5, 9).

*Male*. Body length 8.0–11.0 mm. OOL=1.5 POD; POL=2.3 POD; MS=1.7 POD. Body punctation very similar to female (Figs 14–22). Colouration similar to female (Figs 14–22), but hind femur with basal yellow mark usually separated from apical mark (Fig. 22); T4 with yellow transverse band posteriorly and broader than band on T5 (Fig. 18); epipygium black (Figs 14, 18).

##### Variation.

Colouration of female: anterior yellow transverse stripe of pronotum sometimes turns to two to six obscure yellow spots, posterior yellow transverse stripe sometimes triangular; pale yellow patch on mesoscutum sometimes absent; yellow lunate mark on hind femur varies in length; T4 sometimes with obscure yellow band (much narrower than that on T5) or a pair of obscure lateral patches. Colouration of male: anterior yellow transverse stripe of pronotum sometimes absent; hind femur sometimes subbasally with yellow lunate mark from base crossing to dorsal border; yellow spots on T1 sometimes minute or absent; T4 sometimes with obscure anterior transverse yellow stripe; metasomal sternite 4 rarely with a pair of quadrate yellow spots; epipygium sometimes with small yellow spot medio-posteriorly.

##### Biology.

Specimens are often collected around old adobe houses (Figs 23–27). Parasitoids of Megachilidae and Anthophoridae, but also Vespidae-Eumeninae and Sphecidae s. str. are reported (Iwata 1933; Habu 1962; Bouček 1974a).

##### Distribution.

China (Beijing, Guangdong, Guangxi, Guizhou, Hebei, Henan, Hong Kong, Hubei, *Hunan, Jiangsu, Jiangxi, Shaanxi, Shanghai, Shanxi, Sichuan, Taiwan, Yunnan, Zhejiang), India, Japan, Korea, Nepal, Russia (Nikols’kaya 1960; Habu 1962; Bouček 1974a; Bouček and Narendran 1981; Narendran 1986; He et al. 2004; Shen 2014).

#### Leucospis yasumatsui

Taxon classificationAnimaliaHymenopteraLeucospidae

Habu, 1961

Figs 28–29 , 30–33

Leucospis yasumatsui Habu, 1961: 83; Bouček 1974a: 196.

##### Type material.

Holotype, ♀ (KYUN), “[CHINA, Shansi,] 6.9”, “Holotype *Leucospis yasumatsui* Habu”.

##### Diagnosis.

**Figures 28, 29.**
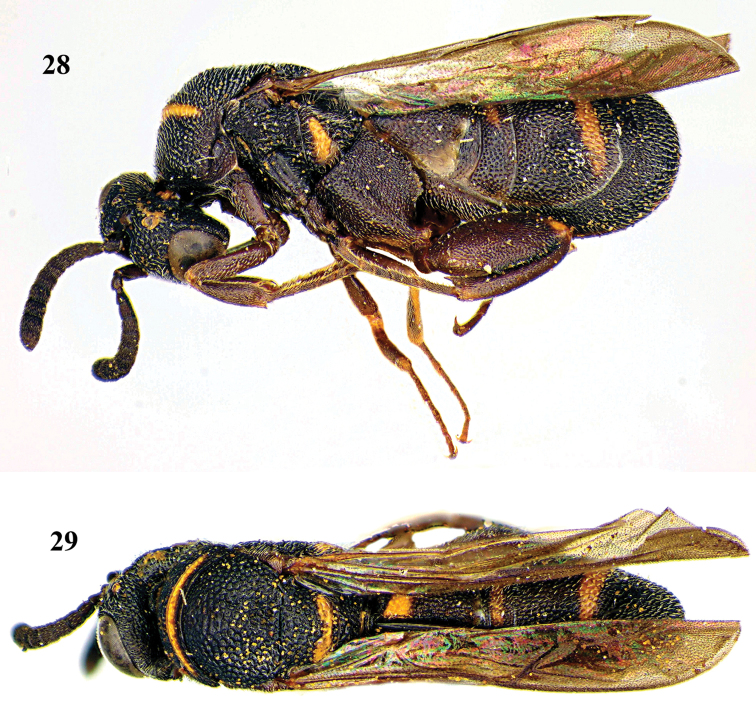
*Leucospis yasumatsui* Habu, ♀, holotype. **28** Habitus lateral **29** habitus dorsal (courtesy of Dr. Toshiharu Mita, Kyushu University).

*Female*. Body mainly blackish brown (Figs 28, 29), with exception of one long yellow transverse band at premarginal carina of pronotum, mesoscutellum with curved yellow transverse band posteriorly, metapleuron with yellow stripe, wings brownish, hind femur with small yellow spot apico-dorsally, T1 with two yellow spots laterad of ovipositorial furrow, T4 with one obscure yellow band anteriorly, T5 with one broad yellow band subposteriorly (Figs 28, 29); pronotum with indistinct discal carina and distinct premarginal carina (Fig. 31); hind femur with ten teeth ventrally, basal tooth largest (Fig. 33); T1 with single ovipositorial furrow; ovipositor reaching anterior margin of T1 (Fig. 29). *Male*. Unknown.

**Figures 30–33.**
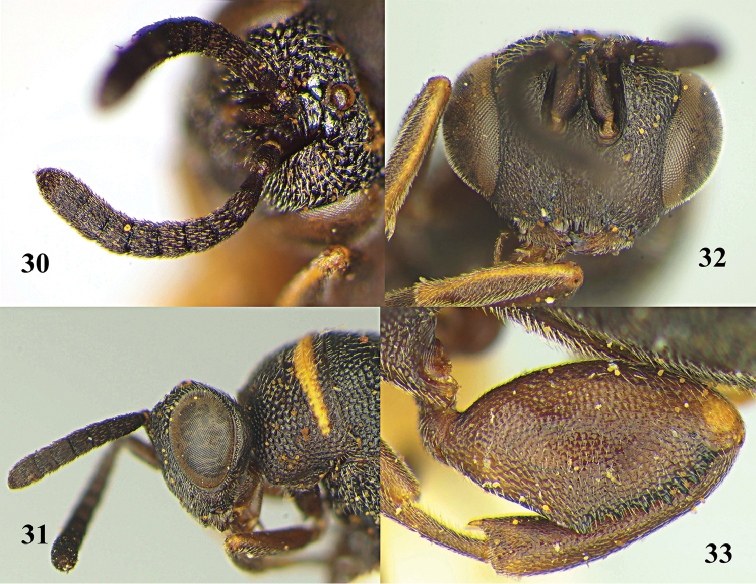
*Leucospis yasumatsui* Habu, ♀, holotype. **30** Antenna **31** head lateral **32** head frontal **33** hind femur and tibia (courtesy of Dr. Toshiharu Mita, Kyushu University).

##### Biology.

Unknown.

##### Distribution.

China (Shanxi), Russia (Storozheva 1986).

### The *elegans*-group

**Diagnosis.** Pronotum with discal, marginal, and premarginal carinae distinct, and strongly angulate and recurved toward mesoscutum; hind femur robust, length at most twice maximum width (Darling and Cardinal 2005).

#### Leucospis aequidentata sp. n.

Taxon classificationAnimaliaHymenopteraLeucospidae

http://zoobank.org/E2F6C63F-1F52-4BE7-90AF-1D80F265E3F8

Figs 34 , 35–42 , 43 , 44–51 , 79

##### Type material.

Holotype, ♀ (SCAU), CHINA, “Guangdong, Shaoguan, Chebaling, 22–28.VII.2008, Zai-fu Xu, No. 2016000345”. Paratypes: 1♀, CHINA, Guangdong, Shaoguan, Chebaling, 22–28.VII.2008, Zai-fu Xu, No. 2016000372 (SCAU); 20♀, CHINA, Hunan, Hupingshan National Nature Reserve, 10–13.VII.2009, Qi Yang, No. 2016000196–2016000199, 2016000201–2016000215 (SCAU, RMNH); 12 ♀, CHINA, Hunan, Hupingshan National Nature Reserve, 10–13.VII.2009, Qing-hui Yi, No. 2016000067 (BMNH), 2016000068 (BMNH), 2016000070 (BMNH), 2016000072, 2016000075, 2016000117, 2016000118, 2016000120, 2016000121–2016000124 (SCAU); 53♀1♂, CHINA, Hunan, Hupingshan National Nature Reserve, 8–12.VII.2009, Shi-hong Wang, No. 2016000085, 2016000088, 2016000090, 2016000091, 2016000097, 2016000098, 2016000100–2016000104, 2016000106–2016000110, 20160001112, 2016000113, 2016000116, 2016000148–2016000151, 2016000153, 2016000155–2016000166, 2016000168–2016000185, 2016000190 (SCAU, KYUN, USNM, ZISP, ZMUC); 5♀1♂, CHINA, Hunan, Hupingshan National Nature Reserve, 8–15.VII.2009, Xin Yuan, No. 2016000078–2016000082, 2016000091 (SCAU); 2♀, CHINA, Hunan, Hupingshan National Nature Reserve, 10–13.VII.2009, 9.VII.2009, Ya-li Tang, No. 2016000126, 2016000127 (SCAU); 4♂, CHINA, Fujian, Minqing, Huangchulin, 13–17.VII.2005, Jing-xian Liu & Li-qiong Weng, No. 2016000043, 2016000045–2016000047 (SCAU, RMNH); 1♂, CHINA, Hubei, Jingshan, 15.VII.2009, Yuan Ye, No. 2016000042 (SCAU).

##### Diagnosis.

**Figure 34.**
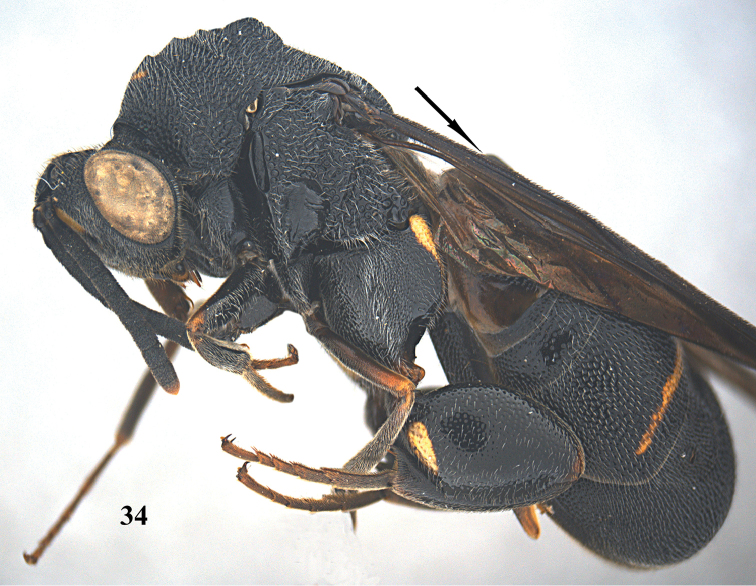
*Leucospis aequidentata* sp. n., ♀, holotype, habitus lateral.

Body mainly black (Fig. 34), with exception of ventrally yellow antennal scape, pronotum with short yellow stripe, wings dark brown, hind coxa with yellow patch baso-dorsally, hind femur with subtriangular yellow patch baso-ventrally, and small obscure reddish yellow spot apico-dorsally, T1 with deep red mark antero-dorsally, T4 with transverse yellow stripe, T5 with transverse yellow stripe (Figs 34, 38, 43, 47); pronotum with strong discal carina and premarginal carina, first one arcuate, latter one straight (Figs 34, 36, 43, 45); dorsellum raised medially and without carinae (Figs 37, 46); hind femur with fourteen teeth ventrally, forming a regular row (Figs 42, 51); concavity below apical spine of hind tibia with rather robust spines and shorter setae (Figs 42, 51, 79); T1 angularly protruding dorsally with deep ovipositorial furrow on top of it and laterally medium-sized smooth interspaces between moderately coarse punctures (Figs 34, 37, 38); dorsal length of T5 0.8 times dorsal length of T1; ovipositorial furrow distinctly impressed on T4 (Fig. 38); ovipositor sheath long, nearly reaching to anterior margin of T1 (Figs 37, 38).

**Figures 35–42.**
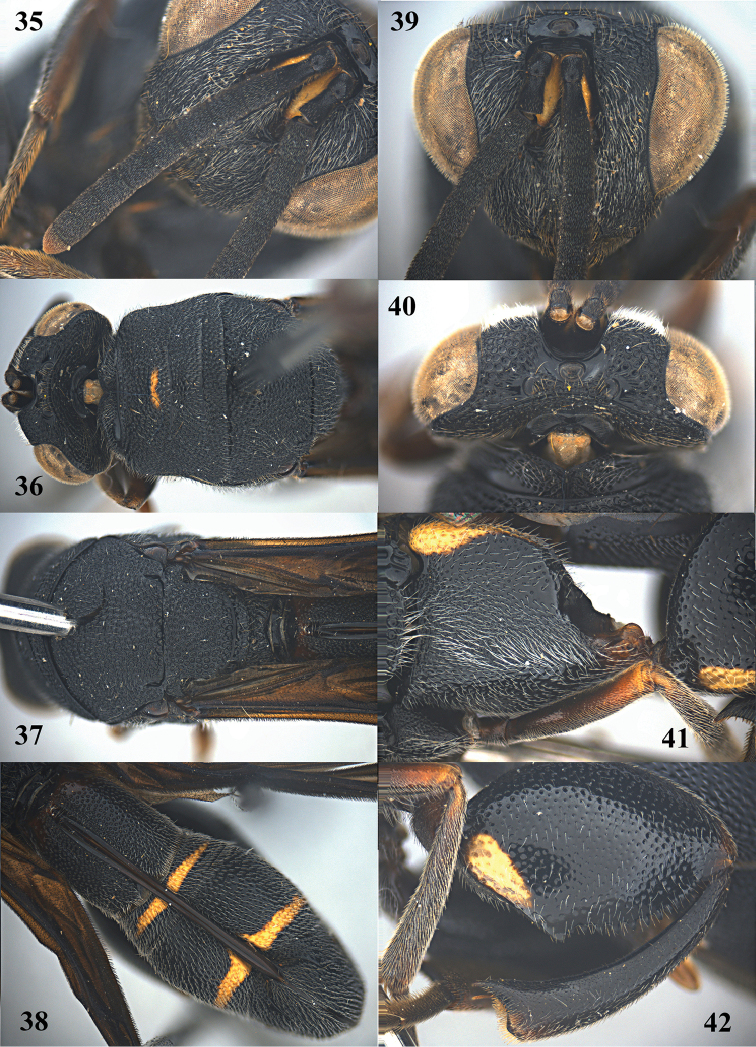
*Leucospis aequidentata* sp. n., ♀, holotype. **35** Antenna **36** head and pronotum dorsal **37** mesosoma dorsal **38** metasoma dorsal **39** head frontal **40** head dorsal **41** hind coxa **42** hind femur and tibia.

**Figure 43.**
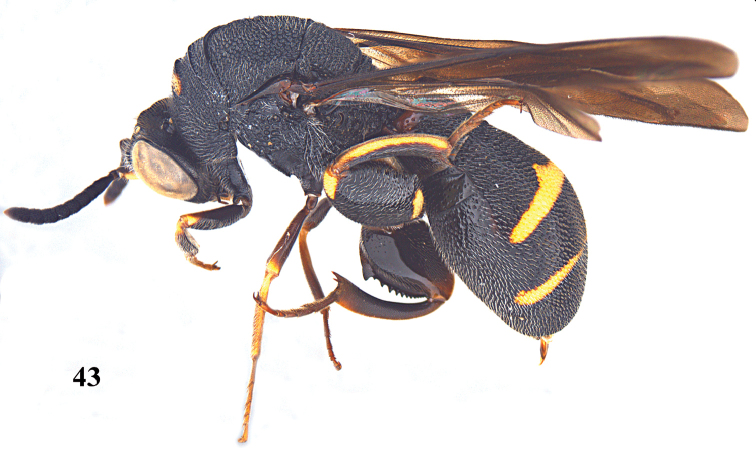
*Leucospis aequidentata* sp. n., ♂, paratype from Hunan, Hupingshan, habitus lateral.

**Figures 44–51.**
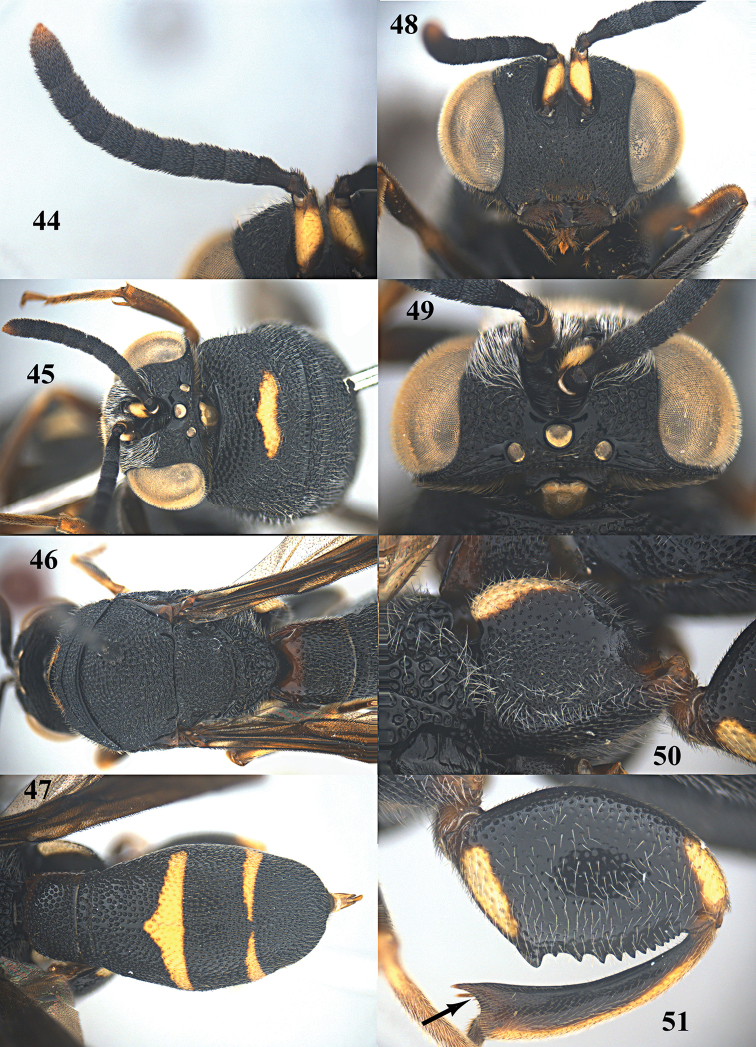
*Leucospis aequidentata* sp. n., ♂, paratype from Hunan, Hupingshan. **44** Antenna **45** head and pronotum dorsal **46** mesosoma dorsal **47** metasoma dorsal **48** head frontal **49** head dorsal **50** hind coxa **51** hind femur.

##### Description.

Holotype. *Female*. Body length 15.0 mm. OOL= 1.4 POD; POL= 2.6 POD; MS= 2.8 POD.

*Head*. Coarsely and densely punctate, with dense and short pubescence (Figs 39, 40). Flagellomeres (except first segment) of female hardly narrowed basally (Figs 34, 35).

*Mesosoma*. Pronotum, mesoscutum, mesoscutellum, dorsellum, mesopleuron, metapleuron and propodeum coarsely and densely punctate, with dense short pubescence (Figs 34, 36, 37). Discal carina and premarginal carina well developed, first one arcuate, latter one straight (Fig. 36). Dorsellum without carina, rounded posteriorly (Fig. 37). Hind coxa moderately punctate (but less densely dorsally and no obvious smooth band), and with short pubescence, carinate postero-dorsally (Fig. 41). Hind femur with regular row of fourteen teeth ventrally, basal one largest (Fig. 42). Hind tibia produced into a spine ventro-apically, concavity below apical spine of hind tibia with rather robust spines and rather short setae (Figs 42, 79). Propodeum with weak median carina.

*Metasoma*. Moderately punctate, with dense short pubescence. T1 narrower than T4 or T5 in dorsal view. Ovipositorial furrow deep on T1, T4 and T5 (Fig. 38). Ovipositor sheath long, nearly reaching anterior margin of T1 (Figs 37, 38).

*Colouration*. Head black (Figs 39, 40). Antenna black, with scape yellow ventrally (Figs 35, 39). Pronotum black, with short and arcuate yellow stripe in front of discal carina (Fig. 36). Mesoscutum, mesoscutellum, dorsellum, mesopleuron, metapleuron and propodeum black (Figs 34, 37). Wings dark brown (Fig. 34). Hind coxa black, with yellow patch baso-dorsally (Fig. 41). Hind femur with subtriangular yellow patch baso-ventrally, and small obscure reddish brown spot apico-dorsally (Fig. 42). Metasoma black, T1 with dark reddish brown mark antero-dorsally, T4 with transverse yellow stripe anteriorly, T5 with transverse yellow stripe posteriorly (Fig. 38); yellow stripe of T5 in lateral view at most about as broad as apical black part of tergite (Fig. 34).

*Male*. Body length 8.0–11.0 mm. OOL= 1.5 POD; POL= 2.3 POD; MS= 1.7 POD. Body punctation and colouration very similar to female (Figs 43–51), but yellow stripe of T5 in lateral view much broader than apical black part of tergite (Fig. 43).

##### Variation.

Colouration of female: sometimes scape yellow entirely, mesoscutellum with transverse yellow patch posteriorly, or a pair of obscure yellow patch posteriorly, hind tibia with yellow patch dorsally. Body length of female: 13.6–17.5 mm. Colouration of male: sometimes scape entirely black.

##### Biology.

Unknown. Collected in July.

##### Distribution.

China (Fujian, Guangdong, Hubei, Hunan).

##### Etymology.

Named after the regular row of teeth of the hind femur.

#### Leucospis femoricincta

Taxon classificationAnimaliaHymenopteraLeucospidae

Bouček, 1974

Figs 52 , 53–60 , 61 , 62–70 , 71–73 , 121–123

Leucospis femoricincta Bouček, 1974a: 184; Bouček and Narendran 1981: 12.

##### Type material.

Holotype, ♀ (BMNH), “VIETNAM, Tonkin, Hoabinh, VIII.1918, R. V. de Salvaza”, “Holotype”, “*Leucospis femoricincta* sp. n., ♀, Z. Bouček det. 1972”, “B.M. Type Hym. 5.2300”, NHMUK010370197. **Additional material.** 1♀, CHINA, Guangdong, Dinghushan National Nature Reserve, 11–12.VIII.2005, Li-qiong Weng, No. 2016000048 (SCAU). 1♀1♂, CHINA, Guangdong, Yingxifenglin, 22.VIII.2016, Qi Yue, No. 2016000129, 2016000130 (SCAU); 1♀, same locality and date, Yi-cheng Li, No. 2016000131 (SCAU); 1♂, same locality and date, Hu-ting Zhou, No. 2016000132 (SCAU); 1♀, same locality and date, Zu-heng Meng, No. 2016002475 (SCAU).

##### Diagnosis.

**Figure 52.**
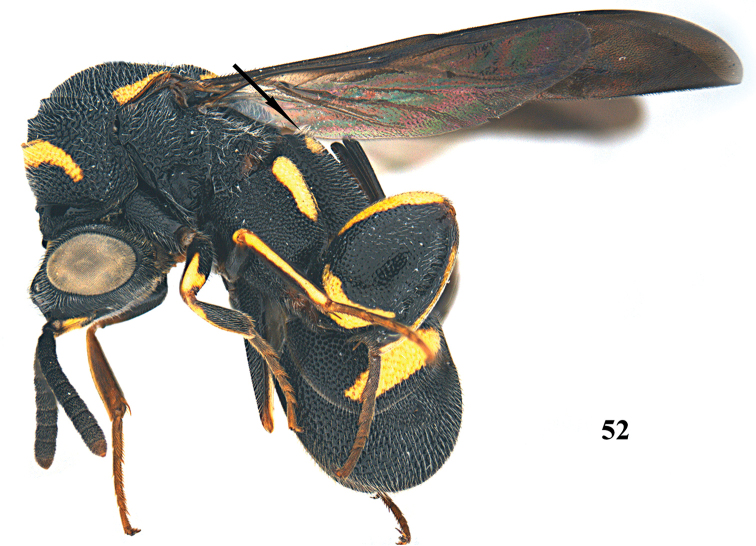
*Leucospis femoricincta* Bouček, ♀ from Guangdong, Yingxifenglin, habitus lateral.

**Figures 53–60.**
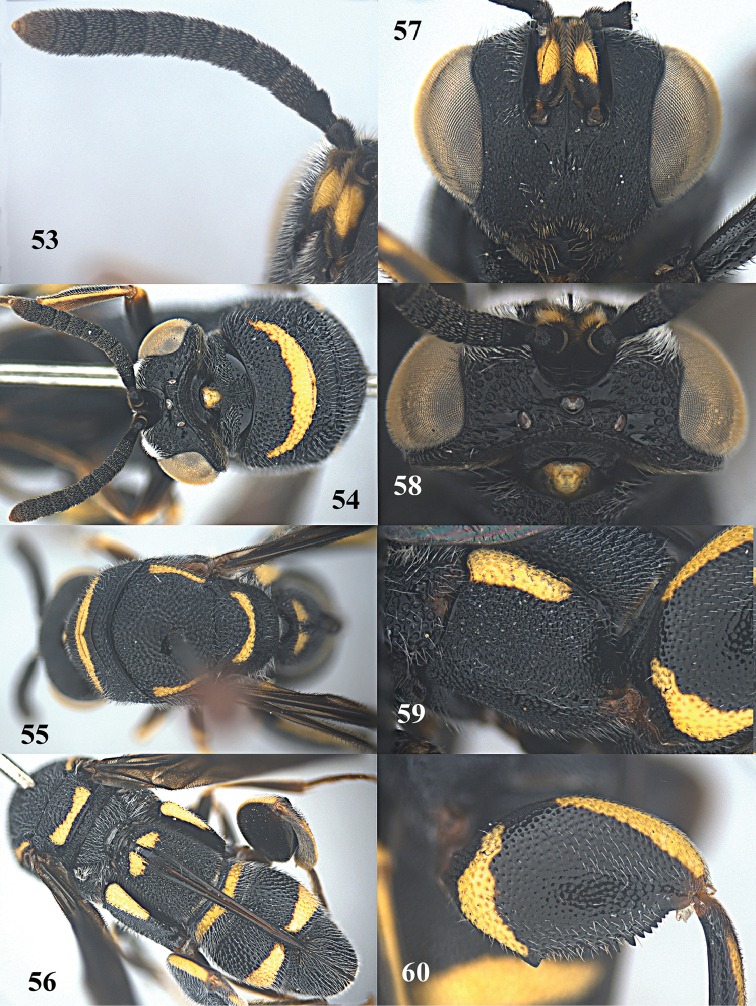
*Leucospis femoricincta* Bouček, ♀ from Guangdong, Yingxifenglin. **53** Antenna **54** head and pronotum dorsal **55** mesosoma dorsal **56** propodeum and metasoma dorsal **57** head frontal **58** head dorsal **59** hind coxa **60** hind femur.

**Figure 61.**
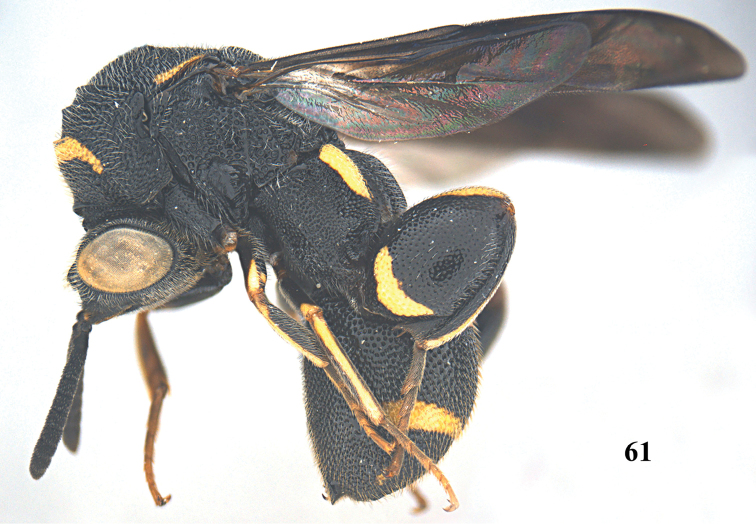
*Leucospis femoricincta* Bouček, ♂ from Guangdong, Yingxifenglin, habitus lateral.

**Figures 62–70.**
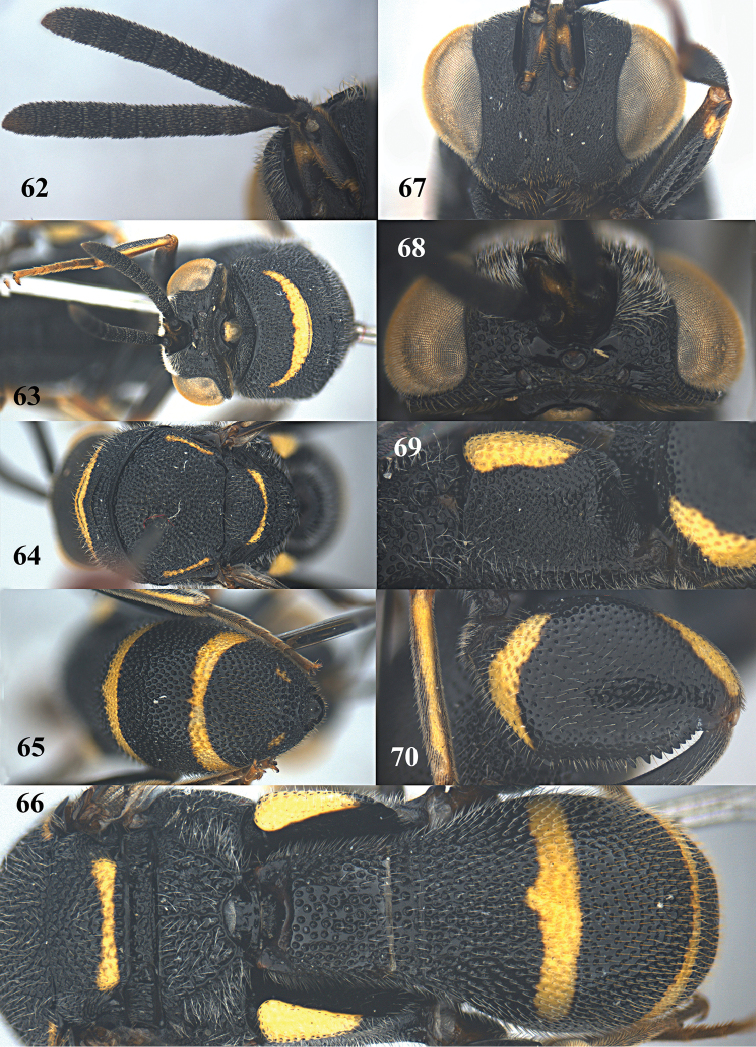
*Leucospis femoricincta* Bouček, ♂ from Guangdong, Yingxifenglin. **62** Antennae **63** head and pronotum dorsal **64** mesosoma dorsal **65** metasoma postero-dorsal **66** propodeum and metasoma dorsal **67** head frontal **68** head dorsal **69** hind coxa **70** hind femur.

Body mainly black (Figs 52, 61), with exception of ventrally partly yellow antennal scape (Figs 53, 57, 62, 67), pronotum with arcuate yellow band (Figs 54, 63), mesoscutum with yellow patch laterally (Figs 55, 64), mesoscutellum with yellow mark posteriorly (Figs 55, 64), wings dark brown, hind coxa with yellow patch baso-dorsally (Figs 59, 69), hind femur with yellow mark subbasally and yellow elongate patch dorsally (Figs 60, 70), T1 with two yellow spots dorsally, T4 with one yellow band anteriorly, T5 with one yellow band posteriorly (Figs 52, 56, 61, 66); pronotum with discal and premarginal carinae distinct, first one much shorter than latter one; propodeum raised medially, with weak median carina; hind femur with eleven teeth ventrally, basal tooth largest (Figs 60, 70); ovipositorial furrow on T1 deep, but shallowly impressed on T4 (Fig. 56); ovipositor sheath long, at least surpassing middle of T1 (Fig. 56).

##### Redescription.

*Female*. Body length 9.4–12.7 mm. OOL= 2.3 POD; POL= 4 POD; MS= 2.3 POD.

*Head*. Coarsely and densely punctate, with dense and short pubescence (Figs 57, 58). Occipital carina developed, sharp. Flagellomeres narrowed basally, F2–F5 broader than long or as long as broad (Figs 53, 54).

*Mesosoma*. Pronotum, mesoscutum, mesocutellum, dorsellum, mesopleuron, metapleuron and propodeum coarsely and densely punctate, with short pubescence (Figs 52, 54, 55). Discal and premarginal carinae distinct, anterior one much shorter than posterior one. Dorsellum rounded and carinae posteriorly. Hind coxa with dense and moderate-sized punctures, and short pubescence, lamellate dorso-posteriorly (Fig. 59). Hind femur finely punctate, with eleven teeth ventrally, basal tooth largest (Fig. 60). Hind tibia produced into a spine ventro-apically (Fig. 52).

*Metasoma*. Coarsely and densely punctate, with short pubescence (Fig. 56). T1 slightly narrower than T4 or T5 in dorsal view. T1 with deep and smooth ovipositorial furrow, this furrow is shallowly impressed on T4 and T5 (Fig. 56). Ovipositor long, at least exceeding half length of T1 (Fig. 56).

*Colouration*. Head black (Figs 57, 58). Antenna black, with scape partly yellow ventrally (Figs 53, 57). Pronotum with one yellow arcuate band at discal carina, not reaching anterior corners (Figs 54, 55). Mesoscutum black, with elongate yellow patch laterally (Fig. 55). Mesoscutellum black, with short U-shaped yellow mark posteriorly (Fig. 55). Dorsellum and propodeum black. Mesopleuron and metapleuron black. Wings brownish. Hind coxa black, with yellow patch baso-dorsally (Fig. 59). Hind femur with lunate yellow mark subbasally, and yellow elongate patch dorsally (Fig. 60). Metasoma black, T1 with two medium-sized yellow spots laterad of ovipositorial furrow, T4 with narrow yellow transverse band, T5 with broad yellow transverse band (Fig. 56). Epipygium black.

*Male*. Body length 8.0–11.0 mm. OOL=1.5 POD; POL=4.8 POD; MS=2.8 POD. Body punctation very similar to female (Figs 61, 63–66). Colouration similar to female, but yellow spots on T1 absent (Fig. 66), yellow transverse band on T4 broader than that on T5 (Fig. 66), epipygium with two small converging yellow spots postero-laterally (Fig. 65).

##### Variation.

One female from Dinghushan has the yellow mark of the hind femur present on the entire dorsal border.

##### Biology.

**Figures 71–73.**
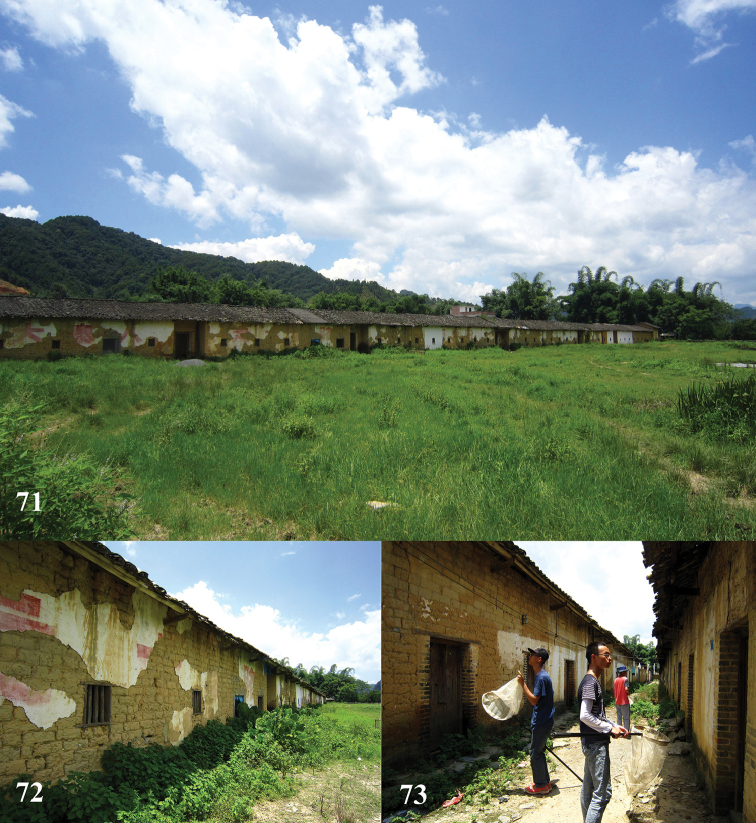
Habitats of *Leucospis femoricincta* Bouček in China.

Unknown. Collected in August, in China near old adobe houses (Figs 71–73).

##### Distribution.

China (Guangdong, Macao), Vietnam (Bouček 1974a).

#### Leucospis shaanxiensis sp. n.

Taxon classificationAnimaliaHymenopteraLeucospidae

http://zoobank.org/955F8699-278D-4D79-89E7-2EC502275C50

Figs 74 , 75–78 , 80–83

##### Type material.

Holotype, ♀ (SCAU), CHINA, “Shaanxi, Liuba, 7.VIII.2015, Yi-cheng Li, No. 2016000020”.

##### Diagnosis.

**Figure 74.**
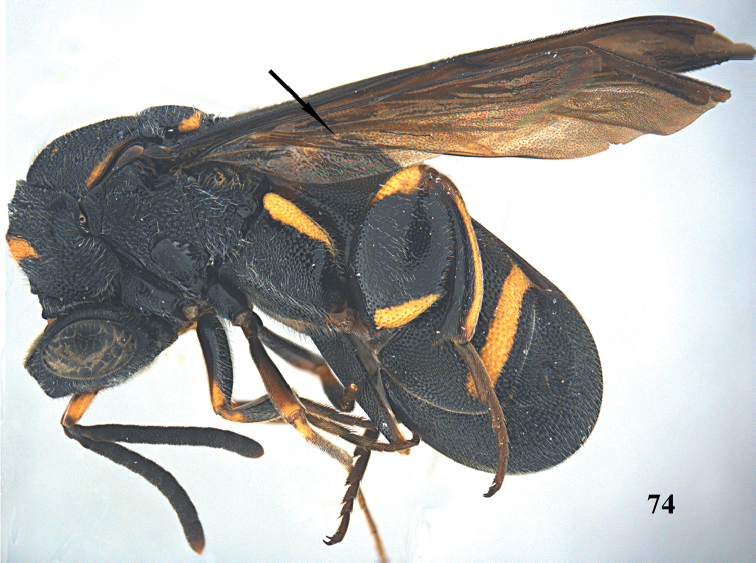
*Leucospis shaanxiensis* sp. n., ♀, holotype, habitus lateral.

**Figures 75–83.**
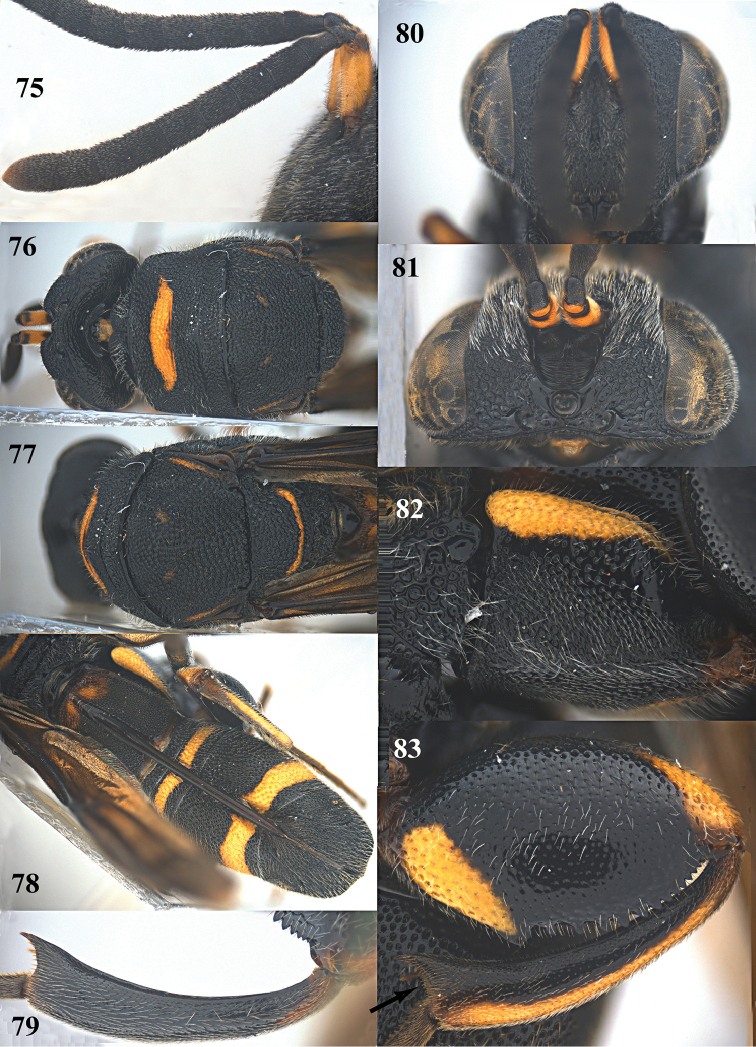
*Leucospis shaanxiensis* sp. n. (but 79 of *Leucospis aequidentata* sp. n.), ♀, holotype. **75** Antenna **76** pronotum and mesoscutum dorsal **77** mesosoma dorsal **78** metasoma dorsal **79** hind tibia **80** head frontal **81** head dorsal **82** hind coxa **83** hind femur and tibia.

Body mainly black (Fig. 74), with exception of largely yellow antennal scape (Figs 75, 80), pronotum with wide yellow stripe (Fig. 76), mesoscutum with a pair of minute yellow spots submedially and a pair of yellow stripes laterally (Fig. 77), mesoscutellum with yellow stripe posteriorly (Fig. 77), wings largely dark brown, hind coxa with yellow stripe dorsally (Fig. 82), hind femur with yellow mark ventro-basally, and similar yellow mark apico-dorsally (Fig. 83), hind tibia mostly yellow dorsally, T1 with a pair of yellowish spots antero-dorsally, T4 with yellow stripe near anterior margin, T5 with yellow stripe near posterior margin (Figs 74, 78); pronotum with distinct discal carina and premarginal carina, first one arcuate, latter one straight; dorsellum without carinae, rounded posteriorly (Fig. 78); hind femur with fourteen teeth ventrally, forming a rather irregular row (Figs 74, 83); concavity below apical spine of hind tibia with slender spines and long setae (Fig. 83); T1 angularly protruding dorsally with ovipositorial furrow on top of it and laterally distinct smooth interspaces between coarse punctures (Fig. 78); ovipositorial furrow on T1 deep, single, and distinctly impressed on T4 (Fig. 78); T5 0.7 times dorsal length of T1 (Fig. 78); ovipositor sheath long, nearly reaching anterior margin of T1 (Fig. 78).

##### Description.

Holotype. *Female*. Body length 15.0 mm. OOL = 2.5 POD; POL = 3.0 POD; MS = 1.9 POD.

*Head*. Coarsely and densely punctate, with dense and short pubescence (Figs 80, 81). Flagellomeres (except first segment) hardly narrowed basally (Figs 74, 75).

*Mesosoma*. Pronotum, mesoscutum, mesoscutellum, dorsellum, mesopleuron, metapleuron and propodeum coarsely and densely punctate, with dense short pubescence (Figs 74, 76–78). Discal carina and premarginal carina well developed, first one arcuate, latter one straight (Fig. 76). Dorsellum without carina, rounded posteriorly (Fig. 77). Hind coxa coarsely and densely punctate (but less densely dorsally), with short pubescence, with narrow smooth area and carinate postero-dorsally (Fig. 82). Hind femur with rather irregular row of fourteen teeth ventrally, basal one largest (Fig. 83). Hind tibia produced into a distinct spine ventro-apically, concavity below apical spine with slender spines and long setae (Fig. 83). Propodeum raised medially, with weak median carina (Fig. 78).

*Metasoma*. Coarsely and densely punctate, with dense short pubescence (Figs 74, 78). T1 little narrower than T4 or T5 in dorsal view (Fig. 78). Ovipositorial furrow deep on T1, T4 and T5 (Fig. 78). Ovipositor sheath long, almost reaching anterior margin of T1 (Fig. 78).

*Colouration*. Head black (Figs 80, 81). Antenna black, with scape yellow but base and apex blackish brown (Figs 75, 80). Pronotum with wide yellow arcuate stripe on discal carina, not reaching anterior corners (Fig. 76). Mesoscutum black, with a pair of minute obscure yellow spots submedially and a pair of narrow yellow stripes laterally (Fig. 77). Mesoscutellum black, with yellow arcuate stripe posteriorly (Fig. 77). Wings dark brown, but medially paler. Hind coxa black, with elongate yellow stripe dorsally (Fig. 82). Hind femur with elongate triangular yellow mark ventro-basally, and similar yellow mark apico-dorsally (Fig. 83). Hind tibia mostly yellow dorsally (Fig. 83). Metasoma black, T1 with a pair of yellowish spots antero-dorsally, T4 with medium-sized yellow stripe near anterior margin, T5 with broad yellow stripe near posterior margin; yellow stripe of T5 in lateral view much broader than apical black part (Figs 74, 78).

*Male*. Unknown.

##### Biology.

Unknown. Collected in August.

##### Distribution.

China (Shaanxi).

##### Etymology.

Named after the province of the type locality.

##### Notes.

Unfortunately, *Leucospis shaanxiensis* is described from a single specimen. We considered the possibility that it is an extreme variant of *Leucospis aequidentata* sp. n., but the number of small differences (both concerning morphology and colouration as indicated in the key) makes this unlikely.

### The *gigas*-group

**Diagnosis.** Pronotum without discal carina, or with very weak discal carina; first and second basal femoral teeth oriented at an angle relative to distal teeth; distal femoral teeth parallel-sided, apices rounded; T5 short, less than 4 times length of T4 (Darling and Cardinal 2005).

#### Leucospis gigas

Taxon classificationAnimaliaHymenopteraLeucospidae

Fabricius, 1793

Figs 84–85 , 86–87

Leucospis gigas Fabricius, 1793: 245; Nikols’kaya 1960: 15; Bouček 1974a: 149; Bouček and Narendran 1981: 5; Narendran 1986: 44; Madl 1989: 200; Madl 1990: 83; 2015: 665; Baur and Amiet 2000: 367; Yildirim et al. 2002: 1187; Lotfalizadeh and Fakhrzadeh 2012: 53; Madl 2014: 796; Madl and Schwarz 2014: 1573.

##### Type material.

Lectotype, ♀ (ZMUC), “FRANCE”, “*Leucospis gigas* Fabricius”, “Z. Bouček det., 1972”, ZMUC00242019, designated by Bouček (1974a).

##### Diagnosis.

**Figures 84, 85.**
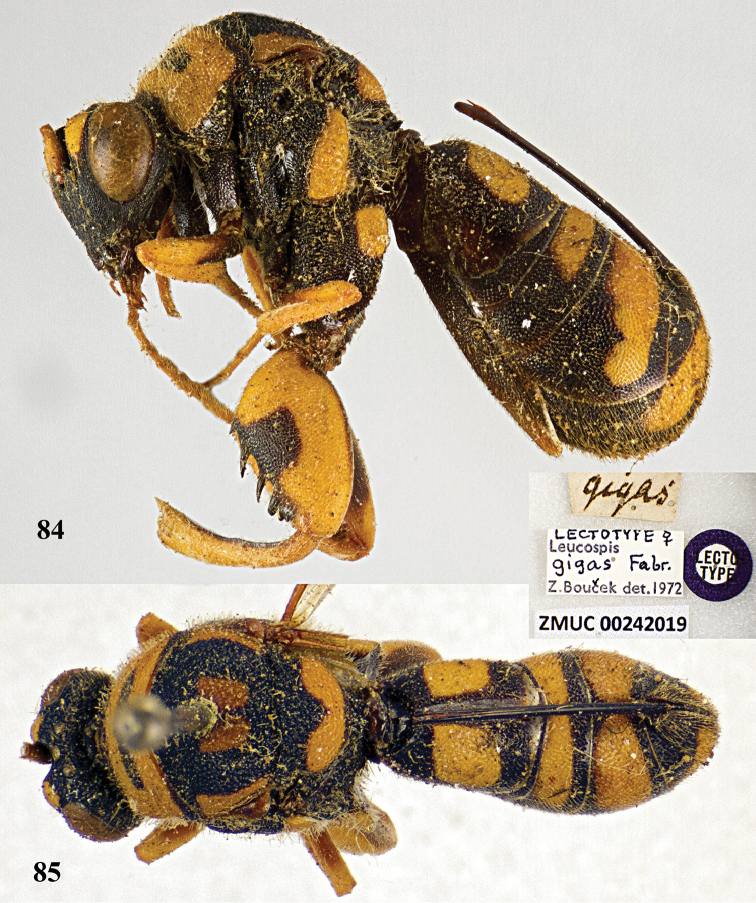
*Leucospis gigas* Fabricius, ♀, lectotype. **84** Habitus lateral **85** habitus dorsal (courtesy of Dr. Lars Vilhelmsen, Natural History Museum of Denmark).

**Figures 86, 87.**
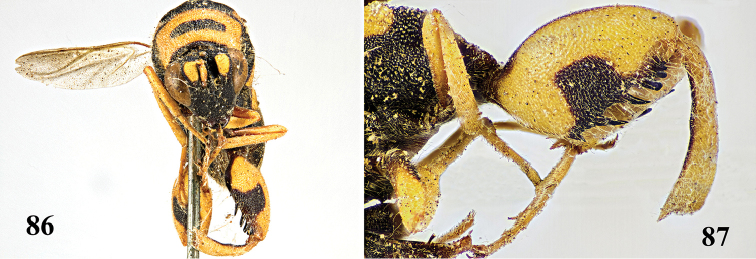
*Leucospis gigas* Fabricius, ♀, lectotype. **86** Habitus frontal **87** legs (courtesy of Dr. Lars Vilhelmsen, Natural History Museum of Denmark).

Body mainly black (Figs 84, 85), with exception of antenna scape yellow, frontovertex with two yellow spots (Fig. 86), pronotum yellow with black transverse band medially (Fig. 86), mesoscutum with a pair of elongate yellow patches laterally, and a pair of yellow spots submedially (Fig. 85), mesoscutellum with yellow U-shaped mark posteriorly, metapleuron yellow (Fig. 85), wings brownish, hind coxa yellow baso-dorsally (Fig. 84), hind femur yellow with quadrate black mark ventrally (Figs 84, 87), T1 with a pair of broad quadrangular yellow marks laterad of ovipositorial furrow (Figs 84, 85); clypeus strongly produced ventrally; flagellum slender, F2–F4 distinct longer than broad; dorsellum bidentate posteriorly; pronotum with distinct premarginal carina; hind femur with seven teeth ventrally, basal tooth short, second and third teeth acute apically, fourth teeth rather obtuse apically (Fig. 87); T1 with ovipositorial furrow (Fig. 85); ovipositor sheath nearly reaching anterior margin of T1 (Figs 84, 85).

*Male*. Not available for this study.

##### Biology.

Parasitoids of Megachilidae and Vespidae (Hymenoptera) (Bouček 1974a; Baur and Amiet 2000; Luo and Liu 2009; Madl and Schwarz 2014). Luo and Liu (2009) gave a detailed report on its life history and oviposition behaviour on its host *Megachile desertorum*. In China collected in June.

##### Distribution.

China (Beijing, Inner Mongolia) (Luo and Liu 2009). Afghanistan, Albania, Algeria, Armenia, Austria, Bosnia-Hercegovina, Croatia, Egypt, France, Germany, Gibraltar, Greece, Hungary, India Iran, Israel, Italy, Jordan, Kazakhstan, Kyrgyzstan, Lebanon, Libya, Macedonia, Malta, Montenegro, Morocco, Pakistan, Romania, Russia, Slovakia, Slovenia, Spain, Switzerland, Syria, Tajikistan, Tunisia, Turkey, Turkmenistan, Ukraine, Uzbekistan (Bouček 1974a; Narendran 1986; Madl 1989, 1990, 2014; Baur and Amiet 2000; Yildirim et al. 2002; Madl and Klimsa 2013; Madl and Schwarz 2014).

#### Leucospis histrio

Taxon classificationAnimaliaHymenopteraLeucospidae

Maindron, 1878

Figs 88 , 89–95 , 96–97

Leucospis histrio Maindron, 1878: cxxx; Bouček 1974a: 164; Bouček and Narendran 1981: 7; Narendran 1986: 44.
Leucospis ornatifrons Weld, 1922: 22. Syn. by Bouček (1974a).

##### Type material.

Holotype of *Leucospis ornatifrons*, ♀ (USNM), “[PHILIPPINES], Manila”, “Type No. 24386, U.S.N.M.”, USNMENT01197921. **Additional material.** 1♀, CHINA, Guangdong, Fogang, Guangyinshan Provincial Natural Reserve, 15–16.IX.2007, Zai-fu Xu, No. 2016000036 (SCAU); 1♀, CHINA, Guangdong, Xiangtoushan National Nature Reserve, 4.VI.2016, Qi Yue, No. 2016000137 (SCAU); 1♀, CHINA, Hainan, Bawangling National Nature Reserve, 21–22.X.2007, Jie-min Yao, No. 2016000041 (SCAU).

##### Diagnosis.

**Figure 88.**
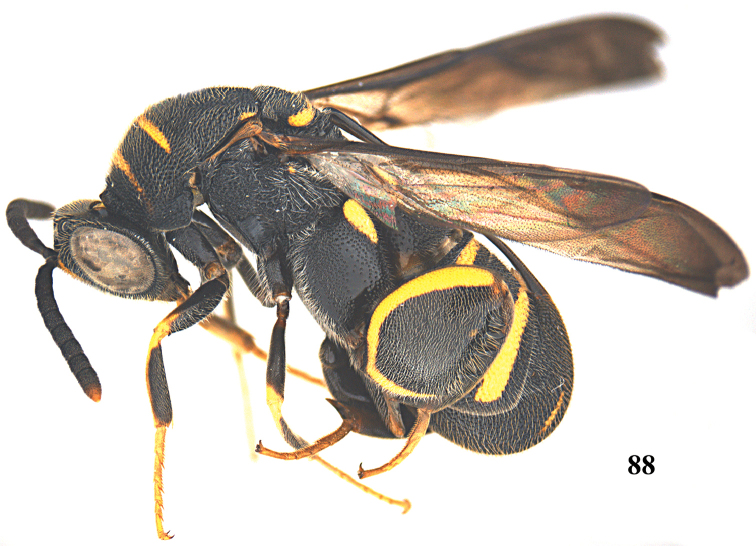
*Leucospis histrio* Maindron, ♀ from Guangdong, Guangyinshan, habitus lateral.

**Figures 89–95.**
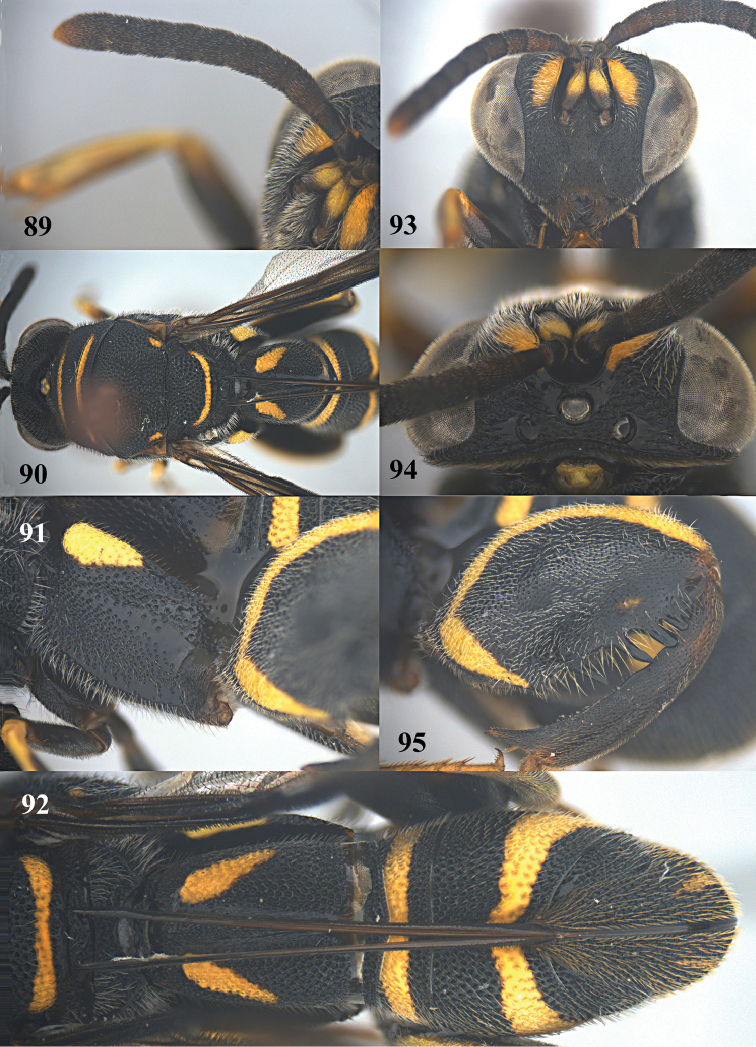
*Leucospis histrio* Maindron, ♀ from Guangdong, Guangyinshan. **89** Antenna **90** mesosoma dorsal **91** hind coxa **92** metasoma dorsal **93** head frontal **94** head dorsal **95** hind femur and tibia.

Body mainly black with yellow pattern (Fig. 88), antennal scape partly yellow ventrally (Figs 89, 93), frontovertex with two yellow spots (Figs 89, 93), wings brownish, hind coxa with yellow patch baso-dorsally (Figs 88, 91), hind femur with yellow mark from base crossing to entire dorsal border and an obscure pale yellow stripe subventrally (Figs 88, 95), epipygium with a pair of slender longitudinal yellow stripes laterally (Figs 88, 92); hind femur with seven teeth ventrally, basal tooth short, second and third teeth acute apically, fourth teeth rather obtuse apically (Fig. 95); T1 with ovipositorial furrow (Fig. 92); ovipositor sheath long, at least reaching dorsellum (Fig. 92).

##### Redescription.

*Female*. Body length 8.5–9.5 mm. OOL= 1.3 POD; POL= 2.8 POD; MS= 2.1 POD.

*Head*. Coarsely and densely punctate (Figs 93, 94). Frons, lower face and clypeus with dense and short pubescence. Clypeus slightly produced ventrally. F2–F5 each distinctly longer than broad (Fig. 89).

*Mesosoma*. Pronotum, mesoscutum, mesoscutellum, dorsellum, mesopleuron, metapleuron, and propodeum with dense, moderate-sized punctures, and medium-sized pubescence (Figs 88, 90, 92). Pronotum with distinct premarginal carina. Dorsellum rounded posteriorly (Fig. 92). Hind coxa finely and densely punctate, with short pubescence, with obvious impunctate band (Fig. 91). Hind femur finely and densely punctate, with short pubescence; with seven tooth ventrally (including three long distinct slender teeth); basal tooth short, second and third teeth acute apically, fourth teeth rather obtuse apically (Fig. 95). Hind tibia produced into a spine ventro-apically (Fig. 95).

*Metasoma*. Metasoma with dense and moderate-sized punctures and short pubescence (Fig. 92). T1 narrower than T4 or T5 in dorsal view (Fig. 92). T1 with double ovipositorial furrow, subdivided by coarsely punctate ridge (Fig. 92). Ovipositor sheath long, at least reaching dorsellum (Fig. 92).

*Colouration*. Head predominantly black, with two elongate yellow spots on frontovertex (Figs 88, 93, 94). Antenna black, with scape partly yellow ventrally (Figs 89, 93). Pronotum black, with two yellow transverse stripes; anterior stripe is similar to and shorter than posterior one (Fig. 90). Mesoscutum black, with a pair of elongate yellow patches laterally, and without small rounded obscure pale yellow spots sublaterally (Fig. 90). Mesoscutellum black, with a curved yellow stripe posteriorly (Fig. 90). Dorsellum and mesopleuron black. Metapleuron black, with a yellow patch. Wings brownish. Fore and mid legs mostly black, with exception of yellowish brown tarsi, and connection between tibia and tarsi yellow. Hind coxa black, with yellow patch baso-dorsally (Fig. 91). Hind femur black, with yellow mark from base crossing to entire dorsal border, and an obscure pale yellow stripe subventrally; hind tibia blackish grown; hind tarsi brown (Fig. 95). T1 black, with two elongate longitudinal yellow marks laterad of ovipositorial furrow (Fig. 92); T2, T3 and T6 entirely black; T4 black, with yellow transverse band anteriorly; T5 black, with yellow transverse band posteriorly; epipygium with a pair of slender longitudinal yellow stripes laterally (Fig. 92).

*Male*. Not available for this study.

**Figures 96, 97.**
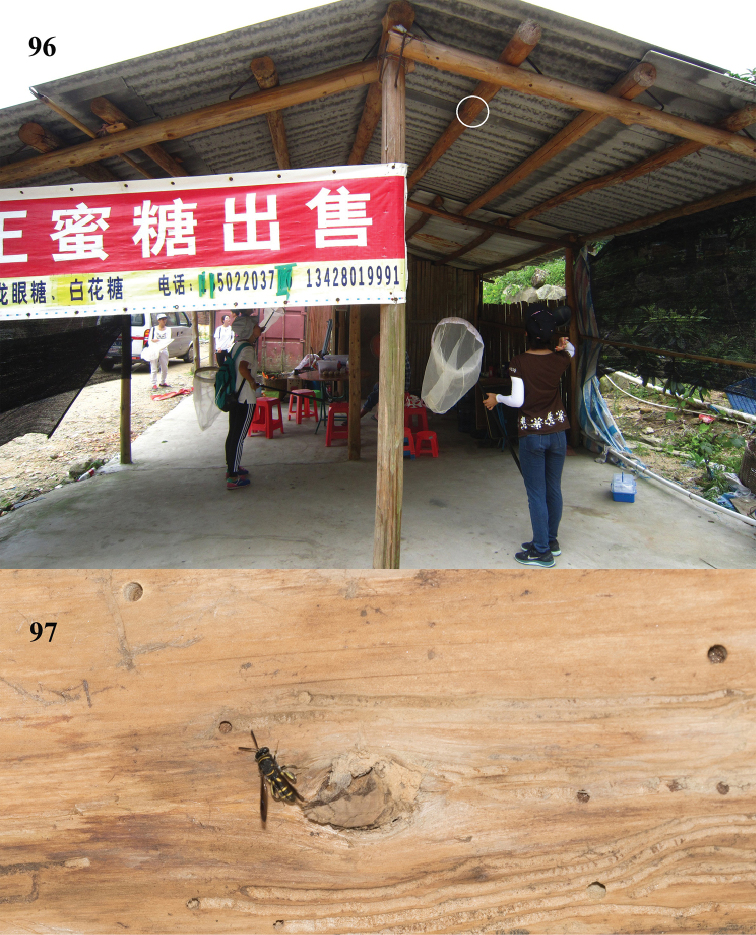
Female *Leucospis histrio* Maindron searching on timber of wooden shed in Guangdong, Xiangtoushan (courtesy of Mr. Wei Dong).

##### Variation.

One female from Xiangtoushan has some different colour patterns: mesoscutum with a pair of short yellow stripes laterally, and a pair of small rounded obscure pale yellow spots submedially; dorsellum mostly black, with a pair of pale yellow spots. Holotype of *Leucospis ornatifrons* has a yellow patch on mesopleuron.

##### Biology.

Parasitoids of Megachilidae and Anthophoridae (Hymenoptera) (Bouček 1974a). Collected in June, September and October in China.

##### Distribution.

China (Guangdong, Hainan), Australia, Bangladesh, India, Malaysia, Myanmar, Papua New Guinea, Philippines, Solomon Islands, Sri Lanka, Thailand (Bouček 1974a; Bouček and Narendran 1981).

#### Leucospis intermedia

Taxon classificationAnimaliaHymenopteraLeucospidae

Illiger, 1807

Figs 98 , 99–105

Leucospis intermedia Illiger, 1807: 130; Bouček 1974a: 153; Bouček and Narendran 1981: 7; Madl 1989: 201; Baur and Amiet 2000: 373; Yildirim et al. 2002: 1188; Madl 2014: 796; 2015: 665; Madl 2014: 796; Madl and Schwarz 2014: 1575.
Leucospis hoplophora Förster, 1851: 17. Syn. by Bouček (1974a).
Leucospis sardoa Costa, 1884: 35. Syn. by Bouček (1974a).
Leucospis sardoa var. minor Costa, 1884: 37. Syn. by Bouček (1974a).

##### Additional material.

1♀, CHINA, Xinjiang, Gongliu, Hetaogou, 29.VI.2016, Yi-cheng Li, No. 2016000128 (SCAU).

##### Diagnosis.

**Figure 98.**
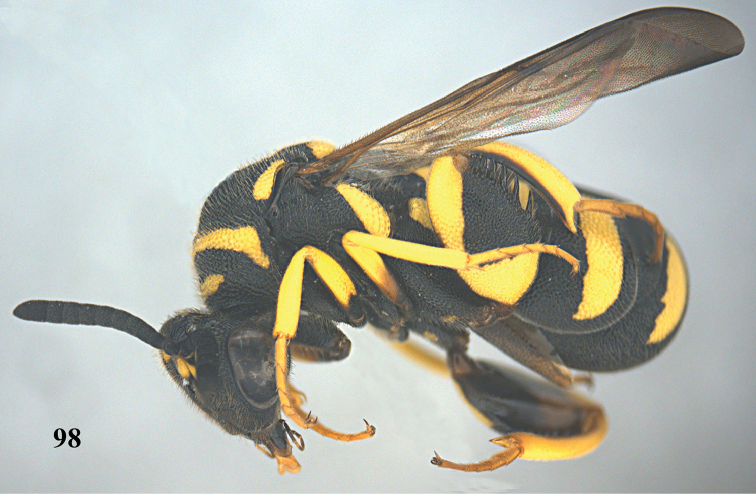
*Leucospis intermedia* Illiger, ♀ from Xinjiang, Gongliu, habitus lateral.

**Figures 99–105.**
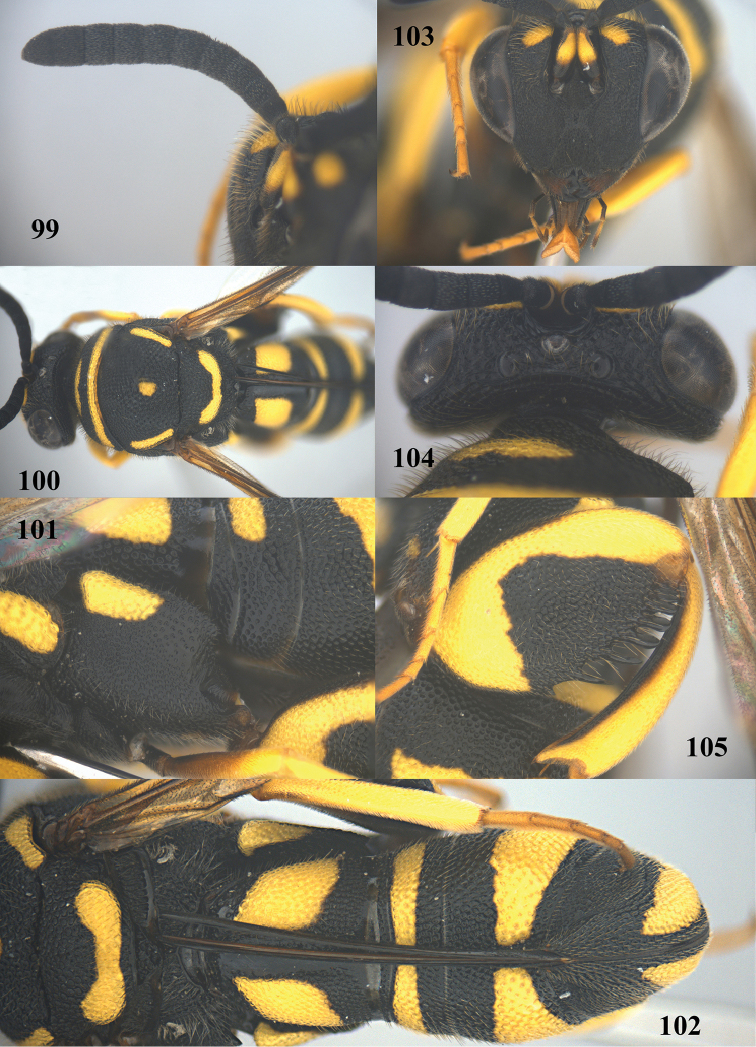
*Leucospis intermedia* Illiger, ♀ from Xinjiang, Gongliu. **99** Antenna **100** mesosoma dorsal **101** hind coxa **102** metasoma dorsal **103** head frontal **104** head dorsal **105** hind femur and tibia.

Body mainly black with yellow pattern (Fig. 98); antennal scape yellow ventrally (Figs 99, 103); head black with two yellow spots on frontovertex (Fig. 103); wings hyaline and infuscated; hind coxa yellow apico-dorsally (Fig. 101), hind femur yellow with triangular black marking ventrally (Figs 98, 105); clypeus produced ventrally (Fig. 103); pronotum with premarginal carina; dorsellum with posterior margin bidentate (Fig. 102); hind coxa without impunctate area (Fig. 101); hind femur with eight teeth ventrally, basal tooth shortest, second tooth acute apically, third and fourth teeth rather obtuse apically (Fig. 105); hind tibia produced into a spine ventro-apically (Fig. 105); T1 with ovipositorial furrow (Fig. 102); ovipositor sheath long, at least reaching anterior margin of T1 (Fig. 102).

##### Redescription.

*Female*. Body length 8.5–9.5 mm. OOL= 1.2 POD; POL= 2.8 POD; MS= 2.8 POD.

*Head*. Coarsely and densely punctate (Figs 103, 104). Clypeus, lower face, and vertex with dense and short pubescence. Clypeus slightly produced ventrally. F2–F11 each broader than long (Fig. 99).

*Mesosoma*. Pronotum coarsely and densely punctate with short pubescence; premarginal carina developed (Fig. 98). Mesoscutum coarsely and densely punctate (Fig. 100). Mesoscutellum coarsely and densely punctate (Fig. 100). Dorsellum coarsely and densely punctate, distinctly bidentate posteriorly (Fig. 102). Hind coxa moderately punctate, without obvious impunctate area, with short pubescence, without tooth on dorsal edge (Fig. 101). Hind femur densely punctate, with eight teeth ventrally (including four long distinct slender teeth); basal tooth shortest, second tooth acute apically, third and fourth teeth rather obtuse apically (Fig. 105). Hind tibia produced into a spine ventro-apically (Fig. 105).

*Metasoma*. Moderately punctate, with short pubescence (Fig. 102). T1 narrower than T4 or T5 in dorsal view (Fig. 102). T1 with double ovipositorial furrow and subdivided by smooth ridge (Fig. 102). Ovipositor sheath long, at least reaching anterior margin of T1 (Fig. 102).

*Colouration*. Body non-metallic (Figs 98, 100, 102). Head predominantly black, with two small yellow spots on frontovertex (Figs 99, 103). Antenna black, with scape yellow ventrally (Fig. 100). Pronotum black, with two yellow transverse stripes (Fig. 100); anterior stripe about half width of pronotum; posterior stripe covers whole width of pronotum (Fig. 100). Mesoscutum black, with a pair of yellow elongate lateral bands and a small yellow median spot (Fig. 100). Mesoscutellum black, with a curved yellow stripe posteriorly (Fig. 100). Dorsellum black. Wings largely infuscated. Propleuron and mesopleuron black. Metapleuron black, with a yellow patch (Figs 98, 101). Fore and mid coxae black; hind coxa black, with yellow stripe antero-dorsally (Fig. 101); trochanters black; fore and mid femora, tibiae and tarsi mostly yellow; hind femur yellow, with triangular black mark ventrally (Fig. 105); hind tibia mostly yellow, with inner margin black (Fig. 105); hind tarsi yellow. Propodeum black. T1 black, with broad yellow mark laterad of ovipositorial furrow (Fig. 102); T2, T3 and T6 entirely black (Fig. 102); T4 black, with yellow transverse band anteriorly (Fig. 102); T5 black, with broad yellow transverse band posteriorly (Fig. 102); epipygium with a pair of longitudinal yellow marks laterally (Fig. 102).

*Male*. Not available for this study.

##### Biology.

Parasitoids of *Osmia emarginata* Lepeletier, *Osmia mustelina* Gerstäcker (Bouček 1974a; Baur and Amiet 2000). In China collected in June.

##### Distribution.

China (Xinjiang) (new record), Afghanistan, Albania, Algeria, Armenia, Austria, Azerbaijan, Caucasus, Croatia, Cyprus, Czech Republic, Egypt, France, Germany, Greece, Hungary, Iran, Israel, Italy, Jordan, Kazakhstan, Lebanon, Libya, Moldova, Morocco, Russia, Slovakia, Spain, Switzerland, Syria, Tadzhikistan, Tajikistan, Turkey, Transcaucasia, Ukraine, Uzbekistan (Bouček 1974a; Madl 2014; Madl and Schwarz 2014; Noyes 2016).

### The *pediculata*-group

**Diagnosis.** Stigmal vein bilobed, stigma and uncus distinct (Darling and Cardinal 2005); hind femur with comb of 25 or more very small teeth or with irregular row of medium-sized teeth (Bouček 1974a).

#### Leucospis bakeri

Taxon classificationAnimaliaHymenopteraLeucospidae

Crawford, 1914

**Figures 106, 107.**
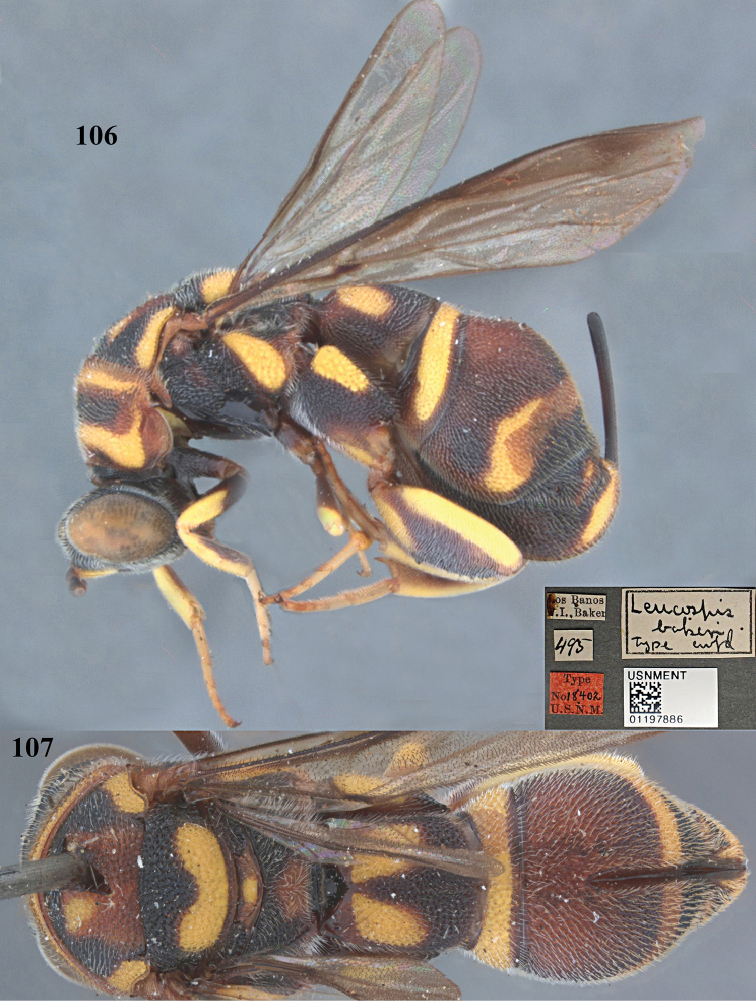
*Leucospis bakeri* Crawford, ♀, holotype. **106** Habitus lateral **107** habitus dorsal (courtesy of Dr. Elijah Talamas, Smithsonian Institution, Washington DC, USA).

**Figures 108, 109.**
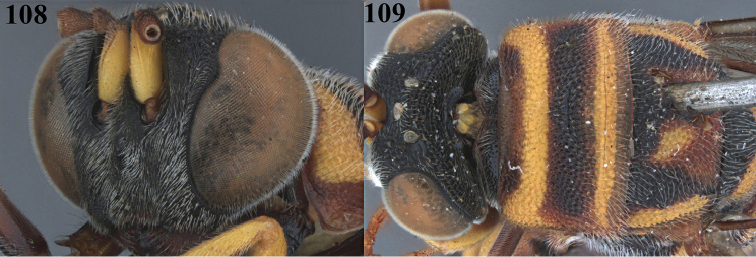
*Leucospis bakeri* Crawford, ♀, holotype. **108** Head antero-lateral **109** pronotum dorsal (courtesy of Dr. Elijah Talamas, Smithsonian Institution, Washington DC, USA).

Figs 106–107 , 108–109

Leucospis bakeri Crawford, 1914: 457; Weld 1922: 30; Bouček 1974a: 198; Bouček and Narendran 1981: 12.
Leucospis gonogastra Masi, 1932: 36. Syn. by Bouček (1974a).

##### Type material.

Holotype of *Leucospis bakeri*, ♀ (USNM), “[PHILIPPINES], Luzon, Los Banos”, “Type No. 18402, U. S. N. M.”, USNMENT01197886.

##### Diagnosis.

Head black (Figs 106, 108, 109); antenna reddish brown, with scape yellow ventrally; mesosoma and metasoma tricoloured (black, yellow and reddish brown) (Figs 106, 107, 109); pronotum with two long yellow transverse stripes (Fig. 109); mesoscutum with a pair of yellow elongate lateral bands and a tricoloured trapezoidal pattern medially (Figs 107, 109); mesoscutellum with a curved yellow mark posteriorly (Fig. 107); dorsellum reddish brown, with yellow spot medially (Fig. 107); metapleuron yellow (Fig. 107); hind coxa with elongate yellow mark baso-dorsally (Fig. 106); hind femur with yellow bands ventro-basally and dorsally (Fig. 106); propodeum reddish brown medio-apically (Fig. 107); T1 with two yellow spots laterally (Fig. 107); T4 with broad yellow transverse band; T5 mainly reddish brown, with yellow transverse band posteriorly; T6 with small yellow spot (Fig. 107); epipygium with a pair of longitudinal marks laterally (Fig. 107); pronotum with weak and short discal carina and distinct and long premarginal carina (Fig. 109); hind coxa with impunctate area, without dorsal teeth (Fig. 106); hind femur with nine medium-sized teeth ventrally, only basal tooth strongest and distinctly protruding (Fig. 106); hind tibia subtruncate apically; metasoma strongly convex dorsally and medially (Fig. 106); T1 without ovipositorial furrow; ovipositor sheath only exceeding half length of T5 (Fig. 107).

*Male*. Not available for this study.

##### Biology.

Unknown. Collected in June and September (Bouček 1974a).

##### Distribution.

China (Taiwan), Philippines (Bouček 1974a).

### The *petiolata*-group

**Diagnosis.** Apex of hind tibia extended into a finger-like projection, outer tibial spur reduced to a short pointed nub; femoral teeth arranged in a line; distal femoral teeth triangular, apices pointed (Darling and Cardinal 2005).

#### Leucospis petiolata

Taxon classificationAnimaliaHymenopteraLeucospidae

Fabricius, 1787

Figs 110 , 111–118

Leucospis petiolata Fabricius, 1787: 285; Bouček 1974a: 174; Bouček and Narendran 1981: 11; Narendran 1986: 45; Sheng 2003: 446.
Leucospis indiensis Weld, 1922: 20. Syn. by Bouček (1974a).

##### Type material.

Lectotype of *Leucospis petiolata*, ♀ (ZMUC), “INDIA, Tranquebar”, “*Leucospis petiolata* Fabricius”, “Bouček det, 1971”, ZMUC00241216, designated by Bouček (1974a). Holotype of *Leucospis indiensis*, ♀ (USNM), “INDIA, Coimbatore”, “28.I.1913”, “Type No. 24384, U. S. N. M.”, USNMENT01223685. **Additional material.** 1♀, CHINA, Guangdong, Nanling National Nature Reserve, 10–14.V.2006, Zai-fu Xu, No. 2016000022 (SCAU); 1♀, same locality, 16–18.X.2007, Zai-fu Xu, No. 2016000023 (SCAU); 1♀, CHINA, Guangdong, Fogang, Guangyinshan Provincial Nature Reserve, 15–16.IX.2007, Zai-fu Xu, No. 2016000037 (SCAU).

##### Diagnosis.

**Figure 110.**
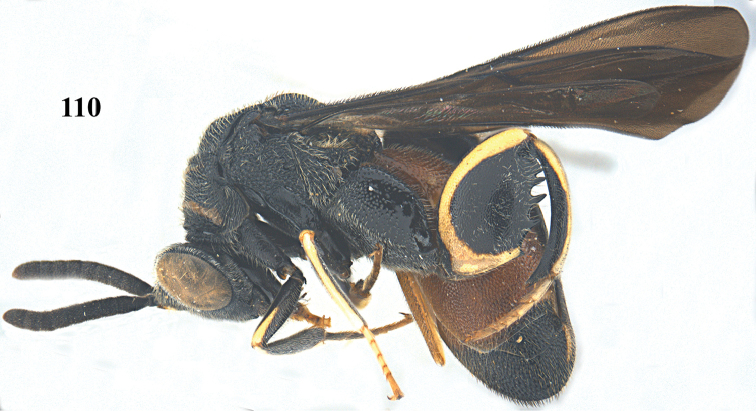
*Leucospis petiolata* Fabricius, ♀ from Guangdong, Nanling, habitus lateral.

**Figures 111–118.**
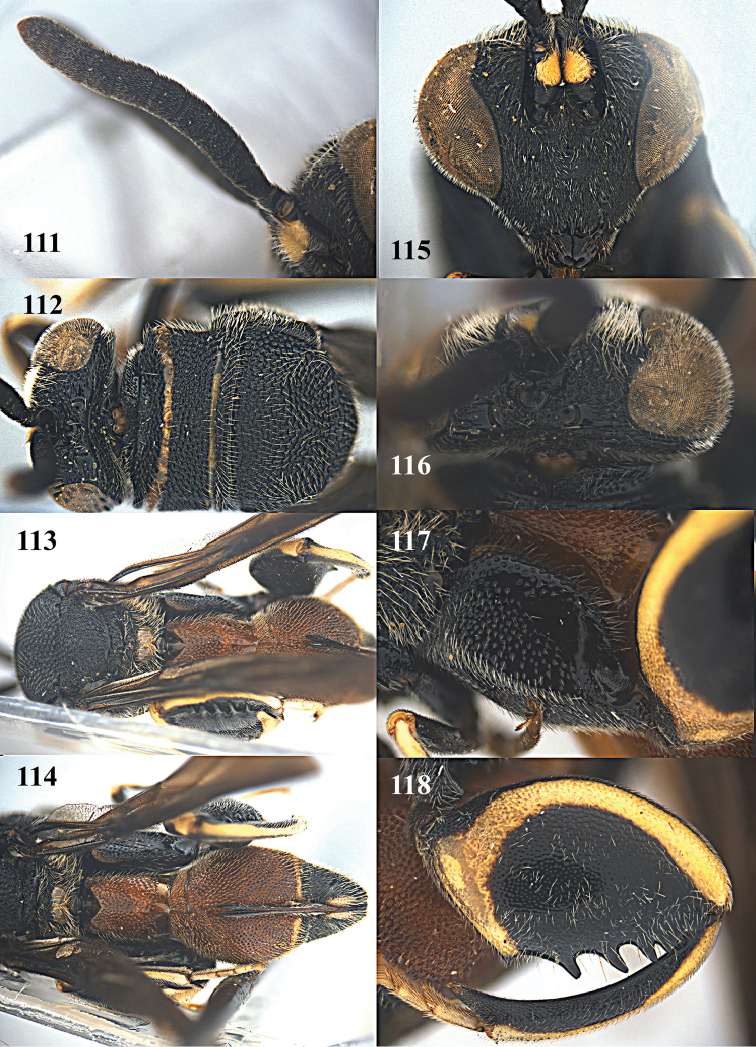
*Leucospis petiolata* Fabricius, ♀ from Guangdong, Nanling. **111** Antenna **112** head and pronotum dorsal **113** mesosoma and metasoma dorsal **114** propodeum and metasoma dorsal **115** head frontal **116** head dorsal **117** hind coxa **118** hind femur and tibia.

Body mainly black or reddish brown with whitish yellow patterns (Figs 110, 112, 114), with antennal scape partly yellowish or ivory ventrally (Figs 111, 115), pronotum with two whitish yellow transverse stripes (Fig. 112), wings brownish, hind femur with whitish yellow mark from base crossing to entire dorsal border, hind tibia mostly whitish yellow dorsally (Figs 110, 118); pronotum with weak discal carina and indistinct premarginal carina; hind femur with nine teeth ventrally, basal one much shorter than following three teeth (Fig. 118); metasoma strongly convex dorsally and medially (Fig. 110); T1 without ovipositorial furrow (Fig. 114); T5 and T6 with ovipositorial furrow (Figs 113, 114); ovipositor sheath only exceeding half-way of T5 (Figs 110, 114).

##### Redescription.

*Female*. Body length 9.6–11.3 mm. OOL= 2.9 POD; POL= 4.0 POD; MS= 3.2 POD.

*Head*. Head with dense short pubescence. Frons, lower face and clypeus moderately punctate (Fig. 115). Vertex coarsely and densely punctate (Fig. 116).

*Mesosoma*. Pronotum, mesoscutum, mesoscutellum, dorsellum, mesopleuron and metapleuron and propodeum coarsely and densely punctate, with short pubescence (Figs 110, 112, 113). Pronotum with weak discal carina, and indistinct premarginal carina replaced by a raised but blunt rib. Dorsellum rounded posteriorly (Fig. 113). Hind coxa finely punctate, with large smooth interspaces and short pubescence, with smooth area dorsally (Fig. 117). Hind femur finely and densely punctate, with nine teeth ventrally; basal tooth much shorter than following three long teeth (Fig. 118). Hind tibia produced into a spine ventro-apically (Fig. 118).

*Metasoma*. Finely punctate, with short pubescence (Fig. 114). T1 much narrower than T4 or T5 in dorsal view (Fig. 114). T1 without ovipositorial furrow (Fig. 114). T5 longer than T1. Ovipositor sheath medium-sized, up to middle of T5 (Fig. 114).

*Colouration*. Head black (Figs 110, 112, 115). Antenna black, with scape partly yellowish ventrally (Fig. 111). Pronotum black, with two slender long whitish yellow and narrowly raised transverse stripes, anterior one arcuate and posterior one straight (Fig. 112). Mesoscutum, mesoscutellum, dorsellum, mesopleuron and metapleuron black (Figs 110, 113). Wings brownish. Fore and mid coxae, trochanters black; fore and mid femora black, with whitish yellow patch apico-dorsally; fore and mid tibiae black, with dorsal border whitish yellow; fore and mid tarsi whitish yellow; hind coxa and trochanter black (Fig. 117); hind femur black, with whitish yellow mark from base crossing to entire dorsal border (Fig. 118); hind tibia black, with dorsal border whitish yellow (Fig. 118); hind tarsi yellowish. Propodeum black, with one whitish yellow transverse spot medio-posteriorly (Fig. 113). T1–T5 reddish brown, sometimes T1 with a pair of whitish yellow spots dorso-laterally, or T5 with whitish yellow stripe posteriorly; T6 black; epipygium black, with a pair of short longitudinal whitish yellow marks laterally (Figs 110, 114).

*Male*. Not available in this study.

##### Biology.

Unknown. In China collected in May and in September–October.

##### Distribution.

China (Fujian, Guangdong, Hong Kong, Macao), Australia, Bangladesh, India, Malaysia, Myanmar, Papua New Guinea, Philippines, Sri Lanka, Thailand, Indonesia (Bouček 1974a).

#### Leucospis sinensis

Taxon classificationAnimaliaHymenopteraLeucospidae

Walker, 1860

Figs 119 , 120

Leucospis sinensis Walker, 1860: 18; Habu 1962: 175; Bouček 1974a: 179; Bouček and Narendarn 1981: 14.

##### Type material.

Lectotype, ♀ (BMNH), “CHINA, Shanghai, Zi-ka-wei”, “B.M. Type Hym. 5.81”, “*Leucospis sinensis* Walker”, “Lectotype”, NHMUK010370188, designated by Bouček (1974a).

##### Diagnosis.

**Figures 119, 120.**
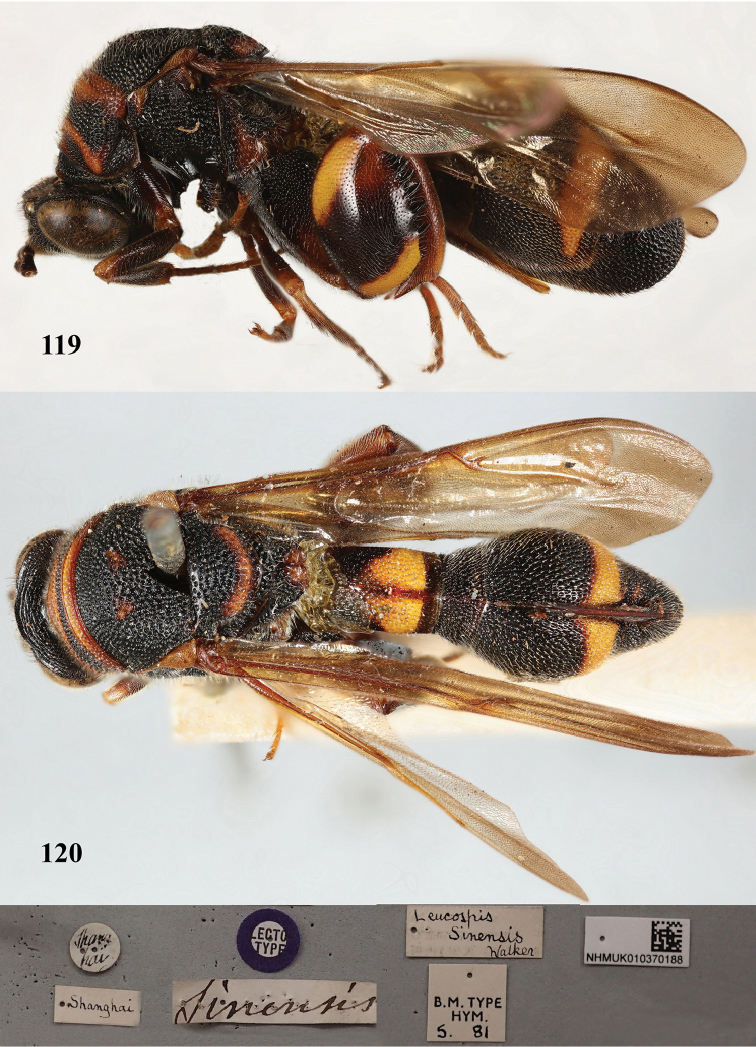
*Leucospis sinensis* Walker, ♀, lectotype. **119** Habitus lateral **120** habitus dorsal (courtesy of Dr. Natalie Dale-Skey Papilloud, The trustees of the Natural History Museum, London, UK).

Body mainly black, with exception of antennal scape partly yellow ventrally, pronotum with two long reddish brown transverse stripes, mesoscutum with two small yellow reddish brown spots submedially, mesoscutellum with curved reddish brown band posteriorly, metapleuron reddish brown, wings brownish, hind coxa reddish brown apically, hind femur with yellow markings ventro-basally and dorso-apically, propodeum with reddish brown spot medio-posteriorly, T1 with broad yellow mark posteriorly, T5 with yellow band posteriorly (Figs 119, 120); pronotum with weak discal carina and distinct premarginal carina; hind femur with nine teeth ventrally, basal tooth much shorter than following six teeth (Fig. 119); hind tibia produced into a spine ventro-apically; propodeum raised medially, with weak median carina; metasoma strongly convex dorsally and medially (Fig. 120); T5 and T6 with ovipositorial furrow (Fig. 120); T1 only carinate medially, without ovipositorial furrow (Fig. 120); ovipositor sheath medium-sized, only reaching half length of T5 (Fig. 120).

*Male*. Not available in this study.

**Figures 121–123.**
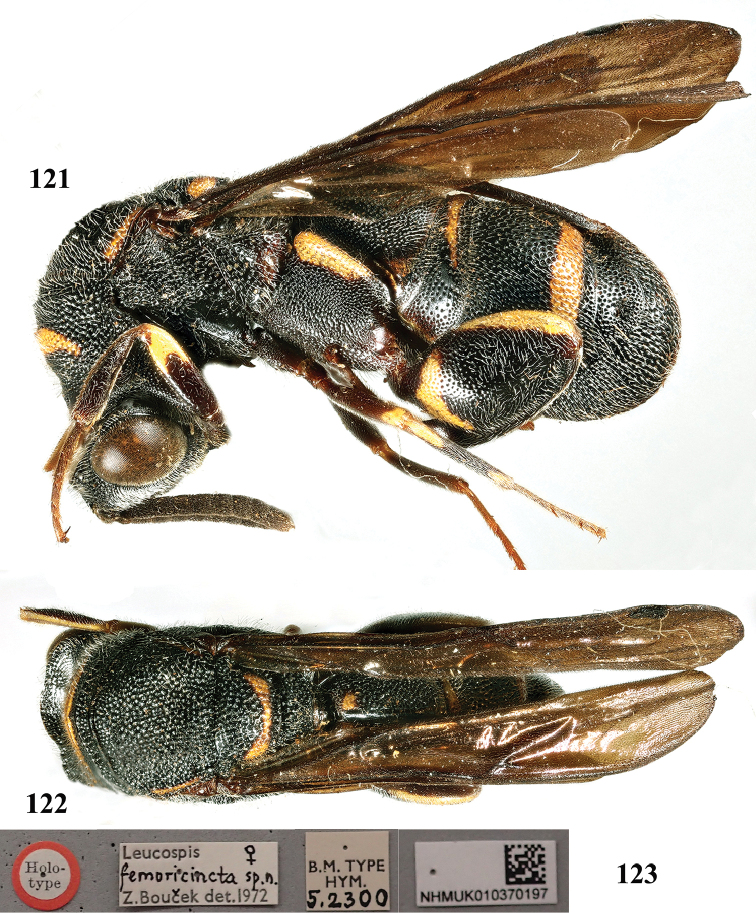
*Leucospis femoricincta* Bouček, ♀, holotype. **121** Habitus lateral **122** habitus dorsal **123** labels (courtesy of Dr. Natalie Dale-Skey Papilloud, The trustees of the Natural History Museum, London, UK).

##### Biology.

Parasitoid of *Sphex nigellus* Smith (Hymenoptera, Sphecidae) (Bouček 1974a).

##### Distribution.

China (Jiangsu, Shanghai, Taiwan), Japan (Bouček 1974a).

## Supplementary Material

XML Treatment for Leucospidae

XML Treatment for Leucospis

XML Treatment for Leucospis aurantiaca

XML Treatment for Leucospis japonica

XML Treatment for Leucospis yasumatsui

XML Treatment for Leucospis aequidentata

XML Treatment for Leucospis femoricincta

XML Treatment for Leucospis shaanxiensis

XML Treatment for Leucospis gigas

XML Treatment for Leucospis histrio

XML Treatment for Leucospis intermedia

XML Treatment for Leucospis bakeri

XML Treatment for Leucospis petiolata

XML Treatment for Leucospis sinensis

## References

[B1] Aguiar AP Deans AR Engel MS Forshage M Huber JT Jennings JT Johnson NF Lelej AS Longino JT Lohrmann V Mikó I Ohl M Rasmussen C Taeger A Yu DSK (2013) Order Hymenoptera Linnaeus, 1758. In: Zhang ZQ (Ed.) Animal Biodiversity: An Outline of Higher-Level Classification and Survey of Taxonomic Richness. Zootaxa 3703(1), 51–62. https://doi.org/10.11646/zootaxa.3703.1.12

[B2] Baur H Amiet F (2000) The Leucospidae (Hymenoptera: Chalcidoidea) of Switzerland, with a key and data on the European species. Revue Suisse de Zoologie 107(2): 359–388. https://doi.org/10.5962/bhl.part.80135

[B3] Bouček Z (1974a) A revision of the Leucospidae (Hymenoptera: Chalcidoidea) of the world. Bulletin of the British Museum (Natural History) Entomology, Supplement 23: 1–241.

[B4] Bouček Z (1974b) Description of a new *Leucopsis* [sic] (Hymenoptera: Leucospidae) from Bolivia. Studia Entomologica 17: 430–432.

[B5] Bouček Z Narendarn TC (1981) The *Leucospis* species of India and adjacent countries (Hymenoptera: Leucospidae). Oriental Insects 15: 1–15. https://doi.org/10.1080/00305316.1981.10434466

[B6] Burks BD (1961) A new Brazilian *Leucospis* parasitic on *Xylocopa*, with a brief review of the South American species of *Leucospis* (Hym., Leucospidae). Studia Entomologica 4: 537–541.

[B7] Cooperband MF Wharton RA Frankie GW Vinson SB (1999) New host and distribution records for *Leucospis* (Hymenopera: Leucospidae) associated primarily with nest of *Centris* (Hymenoptera: Anthophoridae) in the dry forests of Costa Rica. Journal of Hymenoptera Research 8(2): 154–164.

[B8] Costa A (1884) Notizie ed osservazioni sulla geo-fauna sarda. Memoria terza. Risultamento della ricerche fatte in Sardegna nella estate del 1883. Atti dell’Accademia della Scienze Fisiche e Matematiche, Napoli (2) 1(9): 57.

[B9] Crawford JC (1914) New Philippine Hymenoptera. The Philippine Journal of Science (D) 9: 457–458.

[B10] Darling DC Cardinal S (2005) The world species-groups of *Leucospis* (Hymenoptera: Leucospidae) – thirty years later. Acta Societatis Zoologicae Bohemicae 69: 49–64.

[B11] Engel MS (2002) The first leucospid wasp from the fossil record (Hymenoptera: Leucospidae). Journal of Natural History 36: 435–441. https://doi.org/10.1080/00222930110059682

[B12] Fabricius JC (1775) Systema Entomologiae, sistens Insectorum Classes, Ordines, Genera, Species, adiectis Synonymis, Locis, Descriptionibus, Observationibus. Kortii, Flensburgi et Lipsiae, 832 pp.

[B13] Fabricius JC (1787) Entomologia systematica emendata et aucta : Secundun classes, ordines, genera, species, adjectis synonimis, locis, observationibus, descriptionibus. Impensis Christ. Gottl. Proft., Hafniae, 348 pp https://doi.org/10.5962/bhl.title.36532

[B14] Fabricius JC (1793) Entomologia Systematica, 2 Copenhagen and Kiel, p. I–VIII + 1–519.

[B15] Förster A (1851) Eine Centurie neuer Hymenopteren. Vierte und fünfte Dekade. Verhandlungen des Naturhistorischen Vereins der Preussischen Rheinlande und Westfalens, Bonn 8: 17.

[B16] Genaro JA (2012) New species of *Leucospis* (Hymenoptera: Leucospidae) from La Hispaniola, Antilles. Solenodon 10: 81–86.

[B17] Grissell EE Cameron SA (2002) A new *Leucospis* Fabricius (Hymenoptera: Leucospidae), the first reported gregarious species. Journal of Hymenoptera Research 11(2): 271–278.

[B18] Grissell EE Schauff ME (1997) A Handbook of the Families of Nearctic Chalcidoidea (Hymenoptera). (Second edition, revised). Entomological Society of Washington, 1–87.

[B19] Habu A (1961) Chalcididae and Leucospidae from Shansi, North China (Hymenoptera). Mushi 35: 79–86.

[B20] Habu A (1962) II. Family Leucospidae. In: Okada Y Uchida T Kuroda N Yamashina Y (Eds) Fauna Japonica. Biogeographical Society of Japan, Tokyo, 165–177.

[B21] Habu A (1977) A new *Leucospis* species from the Ryukyus, Japan. Entomological Review of Japan 30(1/2): 47–51.

[B22] Hanson PH (1995) Chapter 11.10. Leucospidae. In: Hanson PH Gauld ID (Eds) The Hymenoptera of Costa Rica. Oxford University Press, Oxford, 342–344.

[B23] He JH (Eds) (2004) Hymenopteran Insect Fauna of Zhejiang. Science Press, Beijing, 104–105. [In Chinese]

[B24] Illiger JCW (1807) Fauna Etrusca sistens Insecta quae in provinciis Florentina et Pisana praesertim collegit Petrus Rossius. Mantissae priore parte adjecta, iterum edita et annotatis perpetuis aucta 2, Helmstadii, 130.

[B25] Iwata K (1933) Studies on the nesting habits and parasites of *Megachile sculpturalis* Smith (Hymenoptera, Megachilidae). Mushi 6: 13–15.

[B26] Lotfalizadeh H Fakhrzadeh N (2012) A short review of the family Leucospidae (Hym.: Chalcidoidea) in Iran. Biharean Biologist 6(1): 51–54.

[B27] Luo JF Liu Q (2009) Life history and oviposition behavior of *Leucospis gigas*. Chinese Bulletin of Entomology 46(1): 77–81. [In Chinese]

[B28] Madl M (1989) Zur Kenntnis der paläarktischen *Leucospis*-Arten unter besonderer Berücksichtigung der Fauna Österreichs (Hymenoptera, Chalcidoidea, Leucospidae). Entomofauna 10(12): 197–201.

[B29] Madl M (1990) Beitrag zur Kenntnis der paläarktischen *Leucospis*-Arten unter besonderer Berücksichtigung der Fauna Österreichs (Hymenoptera, Chalcidoidea, Leucospidae). Linzer biologische Beiträge 22(1): 81–87.

[B30] Madl M (2014) New records of the family Leucospidae (Hymenoptera, Chalcidoidea) from Kazakhstan. Linzer biologische Beiträge 46(1): 795–797.

[B31] Madl M (2015) Notes on Palaearctic Leucospidae (Hymenoptera, Chalcidoidea), especially from Libya, Egypt, Iran and Pakistan. Linzer biologische Beiträge 47(1): 665–666.

[B32] Madl M Klimsa E (2013) An aberrant colour form of *Leucospis gigas* (Fabricius, 1793) (Hymenoptera: Chalcidoidea: Leucospidae) from Turkey. Zeitschrift der Arbeitsgemeinschaft Österreichischer Entomologen 65: 122.

[B33] Madl M Schwarz M (2012) Catalogue and faunistics of the family Leucospidae (Hymenoptera: Chalcidoidea) of the Ethiopian region excluding Malagasy subregion. Linzer biologische Beiträge 44(2): 1221–1235.

[B34] Madl M Schwarz M (2014) Notes on Palaearctic species of the family Leucospidae (Hymenoptera, Chalcidoidea), with new records from North Africa and Middle East. Linzer biologische Beiträge 46(2): 1569–1580.

[B35] Maindron M (1878) Descriptions of new *Leucospis*. Bulletin de la Société Entomologique de France 5(8): 109–130.

[B36] Nagase N (2007) Description of a new species of *Leucospis* (Insecta, Hymenoptera, Leucospidae) from the Ogasawara Islands, Japan. Bulletin of the National Museum of Nature and Science (A) 33(1): 4l–44.

[B37] Narendran TC (1986) Family Leucospidae. In: Subba Rao BR Hayat M (Eds) The Chalcidoidea (Insecta: Hymenoptera) of India and the adjacent countries Part II. A Catalogue of Chalcidoidea of India and the adjacent countries. Oriental Insects 20: 43–45. https://doi.org/10.1080/00305316.1986.10433717

[B38] Naumann ID (1981) A new species and additional records of *Leucospis* Fabricius (Hymenoptera: Leucospidae) from Australia. Journal of the Australian Entomological Society 20: 223–228. https://doi.org/10.1111/j.1440-6055.1981.tb01038.x

[B39] Nikols’kaya MN (1960) Chalcididae and Leucospidae in Central Asia (Hymenoptera, Chalcidoidea). Trudy Zoologicheskogo Instituta Akademiya Nauk SSR 27: 220–247.

[B40] Noyes JS (2016) Universal Chalcidoidea Database. http://www.nhm.ac.uk/chalcidoids [accessed 16.04.2016]

[B41] Pauly A Vago JL Wahis R (2003) The apple green colour of Hymenoptera of Madagascar (Vespidae, Apidae, Pompilidae, Leucospidae). Annalen van het Koninklijk Museum van Belgisch-Congo, Tervuren, België, Zoologische Wetenschappen 291: 93–95.

[B42] Pujade-Villar J Caicedo G (2010) Description of a new Colombian species of Leucospidae: *Leucospis vallicaucaensis* n. sp. (Hymenoptera: Chacidoidea [sic]). Dugesiana 17(2): 138.

[B43] Schmid-Egger C (2010) Order Hymenoptera, family Leucospidae. In: van Harten A (Ed.) Arthropod fauna of the United Arab Emirates, Volume 3. Multiply Marketing Consultancy Services, Abu Dhabi, 319–324.

[B44] Shen XC (2014) Insect Fauna of Henan. Science Press, Beijing, 1009–1010. [In Chinese]

[B45] Sheng JK (2003) Leucospidae. In: Huang BK (Ed.) Fauna of Insects in Fujian Province of China. Vol. 7, Fujian Science & Technology Publishing House, Fuzhou, 446–447. [In Chinese]

[B46] Shestakov A (1923) De species nova subspecieque parum cognita generis *Leucospis* F. (Hymenoptera, Chalcididae). Annales du Musée Zoologique. Académie Imperiale des Sciences, Russie 24: 96–100.

[B47] Storozheva NA (1986) *Leucospis yasumatsui* Habu, 1961 (Hymenoptera, Leucospidae) - new species to the USSR fauna. Sistematika i ekologia nasekomyh Dal’nego Vostoka, Vladivostok 1986: 70–72.

[B48] Strand E (1911) Neue und wenig bekannte exotische Arten der Chalcididengattungen *Megastigmus* Dalm., *Mesodiomorus* Strand (n.g.), *Polychromatium* D.T. und *Leucospis* F. Wiener Entomologische Zeitung 30: 93–99.

[B49] Walker F (1834) Monographia Chalciditum. (Continued). Entomological Magazine 2(1): 13–20.

[B50] Walker F (1860) Characters of undescribed species of the genus *Leucospis*. Journal of Entomology 1: 16–23.

[B51] Walker F (1871) Notes on Chalcidiae. Part IV: Chalcididae, Leucospidae, Agaonidae, Eucharidae, Perilampidae, Ormyridae, Encyrtidae. ED Janson, London, 57–70.

[B52] Weld CJ (1922) Studies on chalcid-flies of the subfamily Leucospidinae, with descriptions of new species. Proceedings of the United States National Museum 61(6): 1–43. https://doi.org/10.5479/si.00963801.61-2427.1

[B53] Yildirim E Calmasur O Madl M (2002) Leucospidae of Turkey (Hymenoptera, Chalcidoidea). Linzer biologische Beiträge 34(2): 1185–1189.

